# Carrier‐Free Stimulus‐Responsive Theranostic Nanoprodrug Enabling GSH‐Consumption‐Promoted Chemo‐Photodynamic Cancer Therapy

**DOI:** 10.1002/advs.77484

**Published:** 2026-09-13

**Authors:** Hansheng Huang, Binglin Sui

**Affiliations:** ^1^ Department of Chemistry University of North Dakota Grand Forks North Dakota USA

**Keywords:** cancer treatment, carrier‐free nanoprodrug, chemo‐photodynamic, drug delivery nanosystems, GSH consumption, stimuli‐responsive, synergistic therapy, theranostic

## Abstract

Synergistic chemo‐photodynamic therapy has been proven more effective for cancer treatment compared to traditional chemotherapy and photodynamic therapy alone. Leveraging the characteristic of cancer cells that glutathione (GSH) plays an indispensable role in the development of drug resistance and reactive oxygen species (ROS) neutralization, we developed a carrier‐free GSH‐consuming theranostic nanoprodrug to deeply explore the GSH‐consumption‐promoted synergistic chemo‐photodynamic therapy for boosted cancer treatment. The GSH‐consuming nanoprodrug consists of paclitaxel (PTX) and a boron‐dipyrromethene (BODIPY)‐based photosensitizer, linked through a GSH‐responsive self‐immolating drug linkage; to serve as a control, a non‐GSH‐consuming nanoprodrug consisting of those two moieties linked by a non‐biocleavable linkage was also developed. The carrier‐free nanoprodrugs have a diameter of ∼65 nm and an ultrahigh drug loading content (>70%), and have exhibited highly efficient suppression against tumor proliferation upon light irradiation to trigger their photodynamic effect, more potent than the free PTX drug, because of the synergistic chemo‐photodynamic therapy. Attractively, the GSH‐consuming theranostic nanoprodrug, in comparison with its non‐GSH‐consuming counterpart, exerted extraordinarily powerful therapeutic effects, benefiting from its integrated advantages, including GSH‐activated traceless PTX release, reduced drug resistance, and disabled ROS neutralization of cancer cells due to the consumption of intracellular GSH, resulting in the ultimate eradication of tumors.

## Graphical abstract

Leveraging the critical role of glutathione (GSH) in mediating drug resistance and scavenging reactive oxygen species (ROS) in cancer cells, a carrier‐free GSH‐consuming theranostic nanoprodrug, consisting of a BODIPY photosensitizer and the chemotherapeutic drug paclitaxel linked through a GSH‐consuming drug linker, is developed to deeply explore the GSH‐consumption‐promoted synergistic chemotherapeutic‐photodynamic therapy for boosted cancer treatment with a favorable biosafety profile.

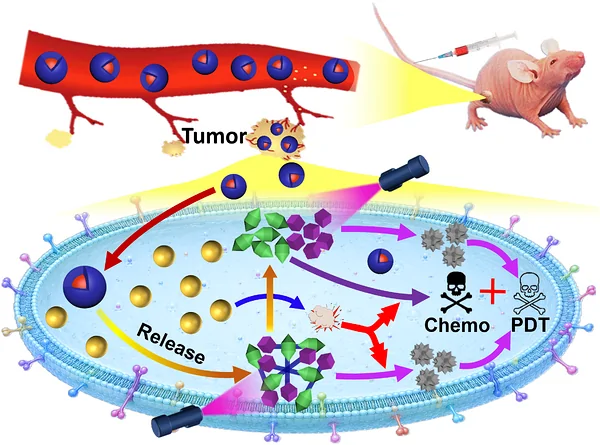

## 1 Introduction

Regarding cancer treatment, conventional chemotherapy is often limited by nonspecific distribution, systemic toxicity, and the development of drug resistance, necessitating advanced therapeutic strategies that improve both specificity and efficacy. Combined treatment modalities, such as chemo‐phototherapy, have attracted considerable interest due to their potential to synergistically enhance tumor eradication through complementary mechanisms of action [1, 2, 3]. Photodynamic therapy (PDT) has emerged as a minimally invasive and highly selective cancer treatment modality that integrates three key components—external light, a photosensitizer (PS), and molecular oxygen—to generate cytotoxic reactive oxygen species (ROS) that induce tumor cell death while sparing healthy tissues [4, 5, 6, 7]. In the synergistic chemo‐photodynamic therapy (chemo‐PDT), cytotoxic chemotherapeutic agents and ROS generated by light‐activated PSs work in concert to induce cancer cell death beyond what either modality can achieve alone [8, 9, 10].

The rapidly evolving nanotechnology has revolutionized drug delivery and therapeutic design, enabling the development of multifunctional nanoplatforms that not only enhance tumor targeting through the enhanced permeability and retention (EPR) effect but also achieve combined diagnostic imaging with therapeutic action [11, 12, 13]. Stimuli‐responsive nanomaterials, which undergo controlled structural or chemical changes in response to internal (e.g., pH, enzymes, redox potential) or external (e.g., light) triggers, have been engineered to achieve on‐demand drug release and enhanced tumor specificity, thereby overcoming several limitations of traditional nanocarriers. Despite those advances, many nanotherapeutic systems rely on inert drug carriers, which can dilute drug loading and raise concerns about long‐term biocompatibility and clearance. Carrier‐free prodrug‐self‐assembled nanostructure, nanoprodrug (NPD), composed entirely of therapeutic agents, has emerged as a promising solution to increase drug payload, improve pharmacokinetics, and reduce off‐target toxicity [14, 15, 16]. These systems exploit molecular interactions such as *π*–*π* stacking and hydrophobic interaction to form stable nanoparticles that can respond to tumor‐specific stimuli to trigger selective drug release [17, 18, 19, 20].

Glutathione (GSH) is a small tripeptide composed of glutamate, cysteine, and glycine, and it serves as one of the most important intracellular antioxidants in living systems. One hallmark of cancer cells is the significantly elevated intracellular concentration of GSH, as they typically experience high levels of oxidative stress due to their rapid proliferation and abnormal metabolism. While normal cells typically contain GSH levels in the micromolar range (approximately 2–20 µm in extracellular fluids), tumor cells often exhibit intracellular concentrations in the millimolar range (about 2–10 mm) [21, 22]. This dramatic increase provides cancer cells with a strong antioxidant defense system, relying heavily on GSH to neutralize ROS, with GSH being oxidized into glutathione disulfide (GSSG). By maintaining redox equilibrium, GSH protects cancer cells from oxidative damage, supports DNA synthesis, and promotes cell survival and proliferation. In addition, high GSH levels are strongly linked to resistance against chemotherapy and radiotherapy. GSH is involved in detoxification pathways that help cancer cells eliminate chemotherapeutic agents through either directly conjugating with anticancer drugs or reducing drug‐induced oxidative stress, thereby decreasing therapeutic efficacy. The pronounced difference in GSH levels in malignant and healthy tissues provides a distinctive biochemical characteristic of the tumor microenvironment. Consequently, over the past two decades, a wide array of GSH‐responsive fluorescent probes and GSH‐activated drug delivery systems have been developed to exploit this disparity, enabling tumor‐selective imaging and controlled drug release with improved therapeutic precision [23, 24, 25, 26, 27]. Disulfide bonds (S─S) are one of the most commonly used redox‐responsive linkages, as they can be selectively cleaved in the presence of elevated intracellular GSH, thereby enabling tumor‐specific activation and regulated drug release [20, 28, 29, 30, 31]. This redox‐triggered behavior not only improves targeting specificity toward tumor tissues but also reduces premature drug leakage and systemic side effects, ultimately leading to enhanced therapeutic efficacy and safety.

Herein, we report the finding that the incorporation of GSH consumption in a theranostic chemo‐PDT nanoprodrug considerably enhanced the therapeutic effect of the synergistic chemo‐photodynamic therapy, taking advantage of the critical role of GSH in mediating drug resistance and scavenging ROS in cancer cells, in addition to the GSH‐triggered tumor‐specific drug release. A GSH‐responsive theranostic prodrug (termed BDP‐SS‐PTX) was designed and synthesized, consisting of a BODIPY‐based photosensitizer and a chemotherapeutic drug paclitaxel (PTX). Boron‐dipyrromethene (BODIPY) dyes represent a class of versatile photosensitizers with attractive photochemical attributes for cancer phototherapy, including tunable absorption/emission wavelengths, high photostability, and the potential for structural modifications that enhance ROS generation. BODIPY derivatives have demonstrated strong singlet oxygen quantum yields and favorable optical properties that can be leveraged for both fluorescent bioimaging and PDT treatment [32, 33, 34, 35, 36, 37, 38, 39], facilitating imaging‐guided theranostic applications [40, 41, 42, 43]. The combination of BODIPY and PTX enables synergistic chemo‐PDT for boosting cancer treatment, attaining high therapeutic payload, tumor‐targeted activation, and elevated antitumor efficacy.

**FIGURE 1:**
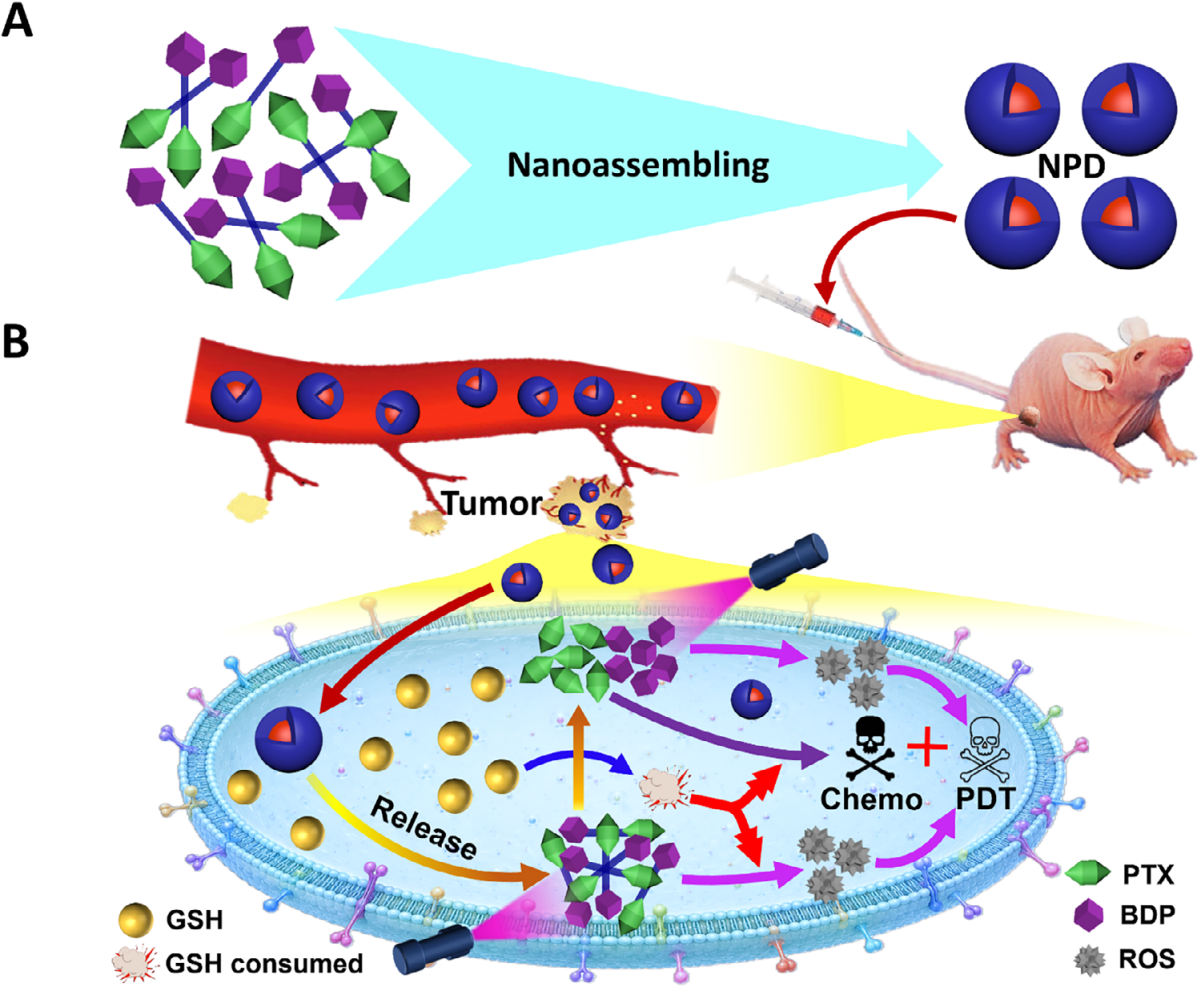
Schematic illustration of (A) the construction of BDP‐SS‐PTX‐NPD and BDP‐PTX‐NPD through a nanoassembling process and (B) the GSH‐consumption‐promoted chemo‐photodynamic therapeutic activities of BDP‐SS‐PTX‐NPD. When administered into the body, the NPDs accumulate in tumor tissues due to the EPR effect. After self‐delivering into cancer cells, the NPDs disassemble into BDP‐SS‐PTX prodrug molecules, which subsequently release PTX molecules through the cleavage of the disulfide bonds, at the consumption of intracellular GSH. Therefore, the intracellular GSH level is downregulated, and consequently, the drug resistance of cancer cells is hampered, leading to elevated chemotherapeutic effect, and their ability to neutralize ROS is dampened, which significantly boosts the therapeutic efficacy of photodynamic therapy. As a result, an overwhelmingly powerful chemo‐PDT therapeutic effect is achieved.

In the BDP‐SS‐PTX molecules, the BODIPY and PTX moieties are linked by a GSH‐sensitive, self‐immolating linker, enabling stimuli‐responsiveness and traceless drug release. To serve as a control, a non‐GSH‐responsive theranostic counterpart of BDP‐SS‐PTX, termed BDP‐PTX, was also synthesized. As depicted in Figure 1A, due to their amphiphilicity, the prodrugs BDP‐SS‐PTX and BDP‐PTX spontaneously assemble into nanoprodrugs, BDP‐SS‐PTX‐NPD and BDP‐PTX‐NPD, respectively, when dispersed into aqueous media. Both NPDs exhibit ultrahigh drug loading content (>70%), benefiting from their carrier‐free feature. Upon self‐delivering into cancer cells, the NPDs disassemble to release their corresponding prodrug molecules. As illustrated in Figure 1B, in the presence of high levels of GSH in cancer cells, the S─S bond of BDP‐SS‐PTX is cleaved, and the self‐immolation of the cleaved drug linker results in the release of intact PTX molecules in a traceless manner, executing chemotherapy. In this process, GSH is consumed, and the downregulation of intracellular GSH inhibits the drug resistance of cancer cells toward PTX, thus enhancing the chemotherapeutic effect of the drug. Subsequently, the external light irradiation triggers the photodynamic effect of BODIPY to generate a large amount of ROS, leading to PDT to kill the cells. As cancer cells rely heavily on GSH to neutralize ROS, the disulfide‐induced GSH inhibition significantly amplifies the photodynamic effect of BDP‐SS‐PTX, in comparison to that of BDP‐PTX. Taken together, by leveraging the dependence of cancer cells on the high‐level intracellular GSH, the theranostic BDP‐SS‐PTX‐NPD achieves potentiated synergistic chemo‐PDT, along with inherent imaging capabilities and GSH‐triggered controlled prodrug activation, providing boosted therapeutic efficacy and offering a promising strategy for improved oncological outcomes.

## 2 Results and Discussion

### 2.1 Molecular Design and Synthesis

**FIGURE 2:**
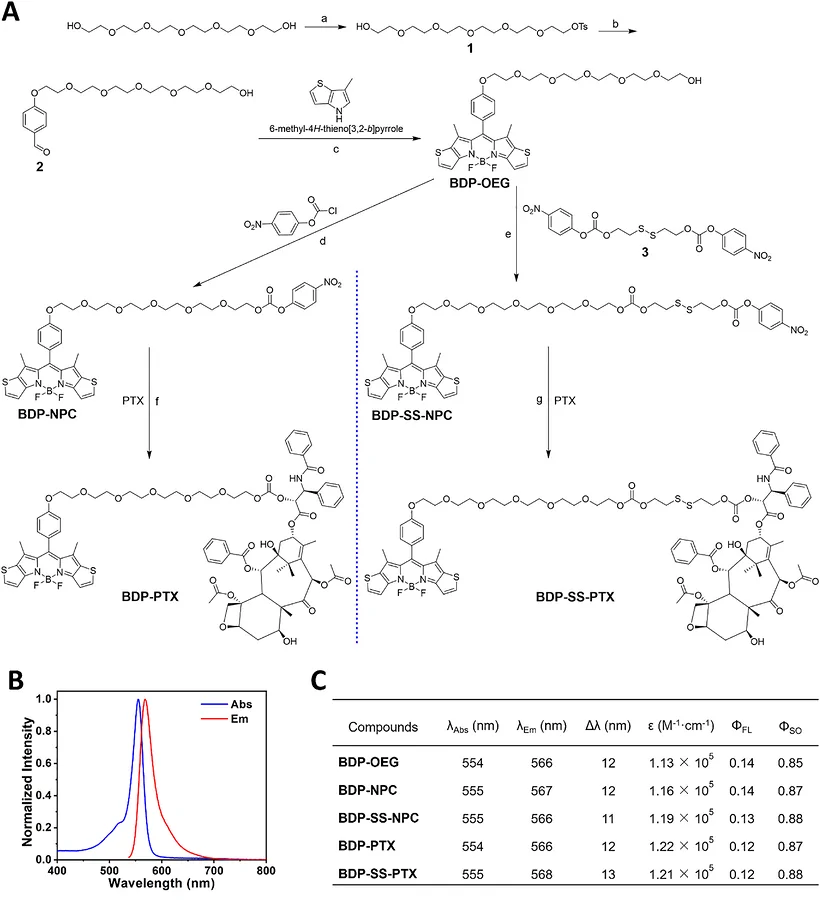
(A) Synthesis of prodrugs BDP‐PTX and BDP‐SS‐PTX. (B) UV–vis absorption (blue) and fluorescence emission (red) spectra of BDP‐SS‐PTX. (C) Photophysical properties of BDP‐OEG, BDP‐NPC, BDP‐SS‐NPC, BDP‐PTX, and BDP‐SS‐PTX, including UV–vis absorption wavelength (λ_Abs_), fluorescence emission wavelength (λ_Em_), Stokes shift (Δλ), molar absorption coefficient (ε), fluorescence emission quantum yield (Φ_FL_), and singlet oxygen quantum yield (Φ_SO_).

The molecular structures of the prodrugs BDP‐PTX and BDP‐SS‐PTX were designed based on the integration of a BODIPY photosensitizer and the chemotherapeutic drug PTX. The two units were linked through a hydrophilic oligoethylene glycol (OEG) chain to balance their hydrophobicity in the prodrug molecules. In addition, a disulfide group was integrated into the OEG drug linker for BDP‐SS‐PTX to equip the prodrug with GSH‐responsiveness. As shown in Figure 2A, the OEG‐contained BODIPY photosensitizer (BDP‐OEG) was synthesized via a condensation reaction between an OEG‐modified 4‐hydroxybenzaldehyde (compound **2**) and a pyrrole derivative, 6‐methyl‐4*H*‐thieno[3,2‐*b*]pyrrole, which was prepared following a reported procedure [44]. Afterward, on one hand, upon reaction with 4‐nitrophenyl chloroformate (NPC), BDP‐OEG was converted to BDP‐NPC, which subsequently reacted with PTX to produce the non‐SS‐contained prodrug BDP‐PTX. On the other hand, compound **3** was synthesized following the synthetic procedure we previously reported [20], and it was used to react with BDP‐OEG to form BDP‐SS‐NPC, which was then converted to the desired GSH‐responsive prodrug BDP‐SS‐PTX via a reaction with PTX. Molecular characterization confirmed the chemical structures of the intermediates and the prodrugs (Figures S1–S16).

### 2.2 Photophysical Characterization

The photophysical properties of BODIPY‐based PSs were systematically investigated using UV–vis absorption and fluorescence emission spectroscopy. They uniformly exhibited a characteristic absorption maximum at 555 nm and a fluorescence emission peak at 567 nm (Figure 2B,C). As summarized in Figure 2C, the PSs have an average fluorescence quantum yield of 0.13, as determined using rhodamine 6G (*Φ*_FL_ = 0.94 in ethanol) as the reference standard, which can be leveraged for biomedical imaging use. Furthermore, the stability of BDP‐SS‐PTX was evaluated under various environmental conditions. The fluorescence intensity of the prodrug remained essentially unchanged across a wide range of pH values (Figure S17) and at different temperatures (Figure S18). In addition, BDP‐SS‐PTX exhibited excellent photostability, as indicated by the minimal change in fluorescence intensity after repeated excitation cycles (Figure S19). This stable and consistent fluorescence response highlights the desirable photophysical robustness of the prodrug and underscores its suitability for bioimaging applications.

### 2.3 Singlet Oxygen Generation Efficiency

**FIGURE 3:**
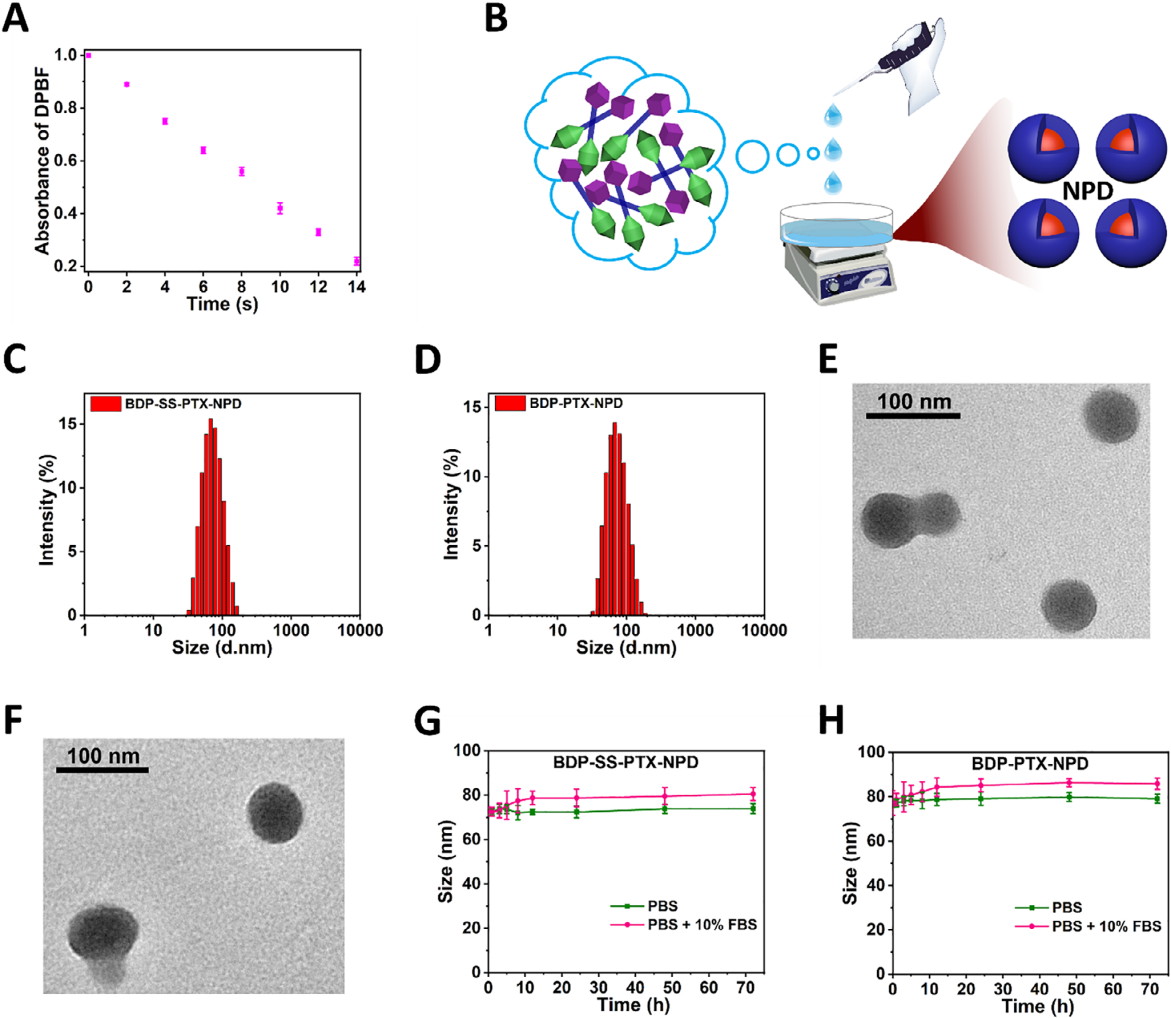
(A) Absorbance of DPBF under continuous light irradiation in the presence of BDP‐SS‐PTX. (B) Fabrication of carrier‐free nanoprodrugs, BDP‐SS‐PTX‐NPD and BDP‐PTX‐NPD via a nano‐assembling procedure. Size distribution of (C) BDP‐SS‐PTX‐NPD and (D) BDP‐PTX‐NPD. TEM images of (E) BDP‐SS‐PTX‐NPD and (F) BDP‐PTX‐NPD. Colloidal and serum stability of (G) BDP‐SS‐PTX‐NPD and (H) BDP‐PTX‐NPD in PBS buffer (pH 7.4) and PBS solutions containing 10% FBS. Data represent the means ± SD, *n* = 3.

The singlet oxygen (^1^O_2_) generation efficiency of BODIPY‐contained compounds was measured in air‐saturated acetonitrile (ACN). Their singlet oxygen quantum yield (*Φ*_SO_) was determined using the commonly used, commercially available sensor 1,3‐diphenylisobenzofuran (DPBF) as an indicator, with rose bengal (RB, *Φ*_SO_ = 0.54 in ACN) employed as the reference standard [45]. As shown in Figure 3A, the absorbance of DPBF gradually decreased under continuous light irradiation in the presence of the PSs, indicating the generation of ^1^O_2_. Based on the linear decay profiles, the ^1^O_2_ quantum yields of the PSs were calculated to be averagely ∼0.87 (Figure 2C). The high ^1^O_2_ generation efficiency indicates their potential as promising theranostic agents for cancer treatment.

### 2.4 Carrier‐Free Theranostic Nanoprodrug Construction

The theranostic nanoprodrugs BDP‐SS‐PTX‐NPD and BDP‐PTX‐NPD were fabricated according to an established nano‐assembling protocol [20]. As described in Figure 3B, when the theranostic prodrugs are dropped into aqueous media, the prodrug molecules spontaneously assemble into nanostructures due to intermolecular *π*–*π* stacking, amphiphilicity balancing, and hydrogen bonding [17, 18, 19, 20]. The drug loading efficiency of BDP‐SS‐PTX‐NPD and BDP‐PTX‐NPD during the self‐assembly process was determined to be 92% and 87%, respectively, and notably, they both exhibited ultrahigh drug loading content (>70%). The assembled NPDs were characterized using dynamic light scattering (DLS) and transmission electron microscopy (TEM). DLS measurements showed that BDP‐SS‐PTX‐NPD and BDP‐PTX‐NPD possessed average hydrodynamic diameters of 72.6 and 77.1 nm, respectively, with a relatively narrow size distribution, as indicated by a polydispersity index of ∼0.21 (Figure 3C,D). TEM analysis further confirmed that the NPDs adopted a uniform spherical morphology with a dry‐state diameter of approximately 65 nm (Figure 3E,F), which is consistent with the DLS results. The particle size of the NPDs falls within an optimal range for tumor‐targeted drug delivery, as nanoparticles with sizes between 5 and 50 nm are generally more efficient at penetrating tumor tissues and entering cancer cells, whereas those in the 50–200 nm range tend to exhibit prolonged blood circulation and enhanced tumor accumulation via the EPR effect [46, 47, 48, 49]. In addition, the NPDs exhibited a negative surface charge, with a zeta potential of approximately −9.5 mV (Figure S20), suggesting adequate colloidal stability.

The hydrodynamic colloidal stability of BDP‐SS‐PTX‐NPD and BDP‐PTX‐NPD was verified in various aqueous environments. When dispersed in deionized water, the nanoparticles showed negligible changes in hydrodynamic size over a period of one week (Figures S21 and S22), indicating excellent stability. To assess their behavior under biologically relevant conditions, the NPDs were incubated in phosphate‐buffered saline (PBS, pH 7.4) in the presence and absence of fetal bovine serum (FBS). Both NPDs maintained consistent hydrodynamic diameters in PBS, and the presence of FBS had minimal impact on particle size (Figure 3G,H). These results demonstrate the high colloidal and serum stability of the NPDs in complex biological media, underscoring their suitability for biomedical applications.

### 2.5 GSH Responsiveness and Drug Release Profiles

**FIGURE 4:**
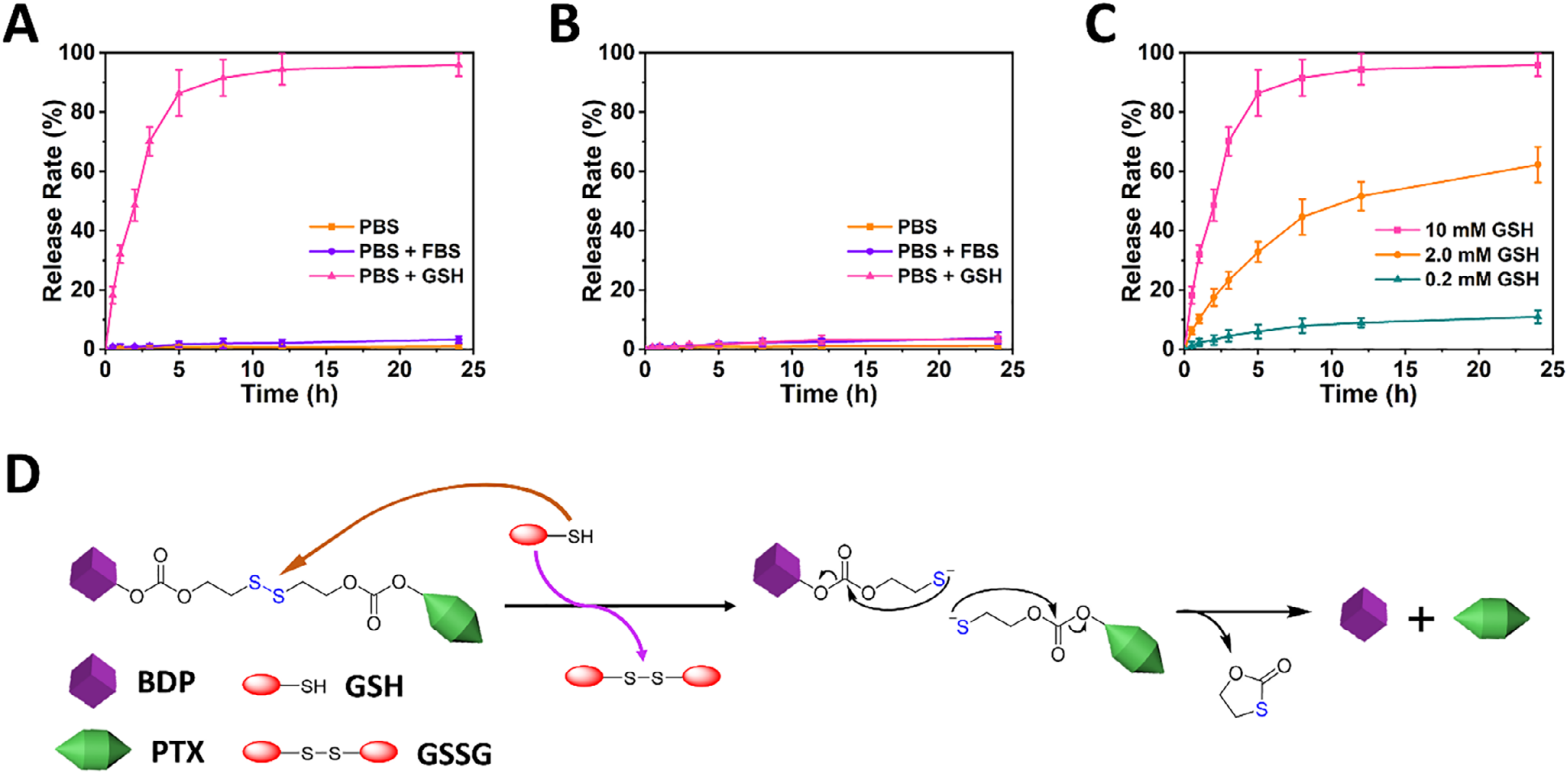
Drug release efficiency of (A) BDP‐SS‐PTX‐NPD and (B) BDP‐PTX‐NPD in PBS buffer and PBS buffer solutions containing 10% FBS or 10 mm GSH. (C) Drug release efficiency of BDP‐SS‐PTX‐NPD in PBS buffer solutions containing different concentrations of GSH. Data represent the means ± SD, *n* = 3. (D) The mechanism of GSH‐triggered traceless release of PTX, with GSH being oxidized to GSSG.

Effective release of PTX molecules is necessary to guarantee the chemotherapeutic effect of the NPDs for treating cancer. The GSH‐responsive drug release profiles of BDP‐SS‐PTX‐NPD were examined in PBS buffer solutions supplemented with or without FBS and GSH, simulating varied biological environments. To serve as a control, BDP‐PTX‐NPD was studied under the same drug release conditions. Both NPDs exhibited sufficient confinement of the loaded PTX molecules in PBS solutions with and without the supplement of FBS (10%), as indicated by the negligible drug release (Figure 4A,B), benefiting from their high dynamic and serum stability, which prevents premature release of drug molecules in blood circulation. However, when GSH (10 mm) was added to the PBS solution of BDP‐SS‐PTX‐NPD, a rapid release of PTX was successfully activated, leading to the release of over 85% of the total loaded drug within 5 h of incubation and approximately 95% within 24 h (Figure 4A). In addition, the release rate of PTX was found to be dependent on the concentration of GSH, as evidenced by the faster drug release in response to higher concentrations of GSH (Figure 4C). These observations confirmed the GSH responsiveness and the GSH‐triggered PTX release of BDP‐SS‐PTX‐NPD. In stark contrast, under the same conditions, the addition of GSH induced no considerable change to the PTX release of BDP‐PTX‐NPD (Figure 4B), indicating the non‐GSH‐responsiveness of the NPD. These findings inferred that the interaction between GSH and the disulfide bond of BDP‐SS‐PTX‐NPD triggered the drug release. As illustrated in Figure 4D, upon reacting with the reducing thiol group of GSH, the disulfide bond is reduced, and simultaneously, GSH is oxidized, being converted into GSSG. The resulting cleaved drug linker self‐immolates into a stable small cyclic molecule, 1,3‐oxathiolan‐2‐one, leading to the traceless release of PTX molecules.

### 2.6 GSH‐Inhibition‐Promoted Synergistic Chemo‐PDT Therapeutic Effect

**FIGURE 5:**
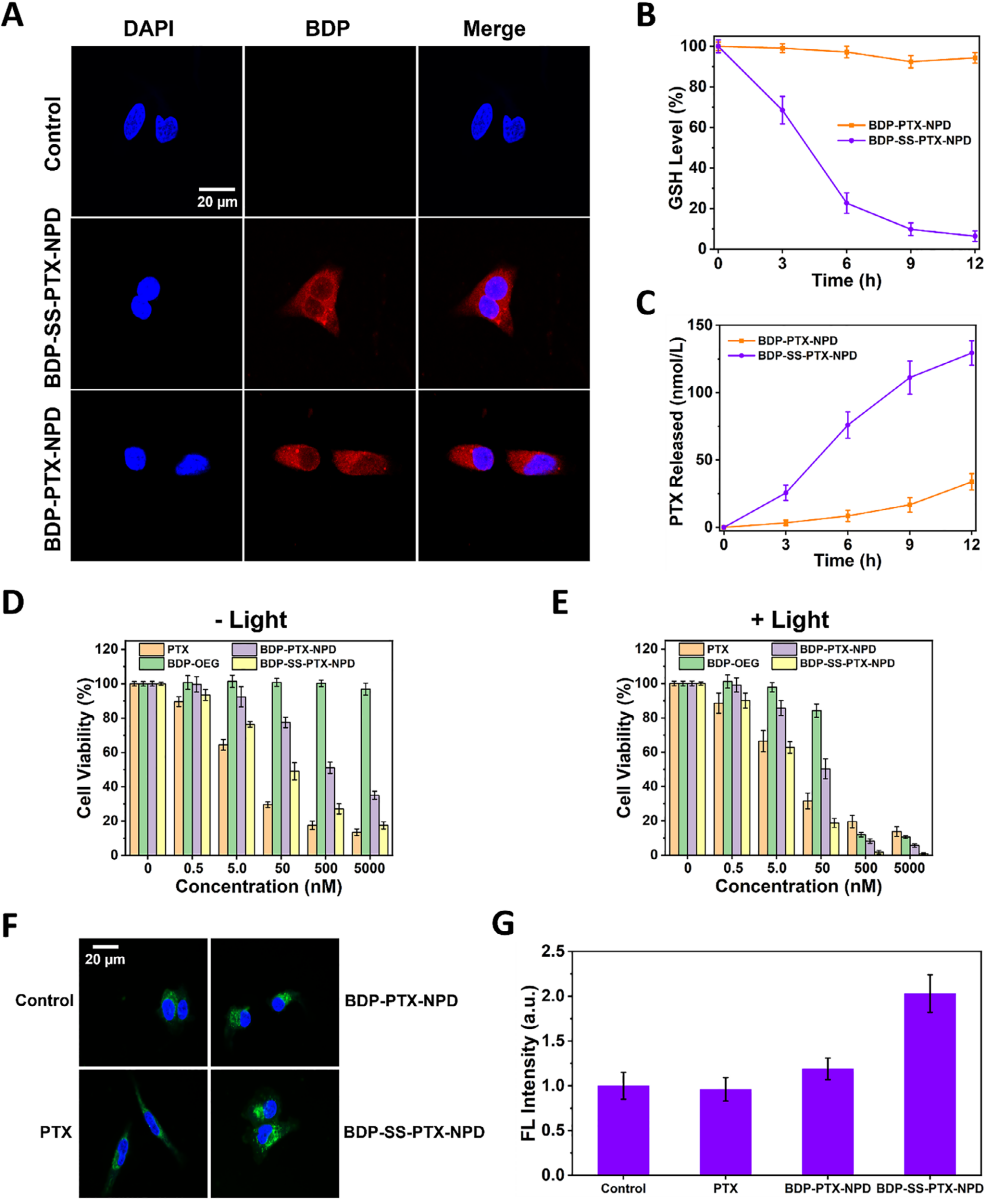
(A) CLSM images of BxPC‐3 cells after incubation with BDP‐SS‐PTX‐NPD and BDP‐PTX‐NPD. The cell nuclei were stained with DAPI. (B) Time‐dependent dynamic level of GSH and (C) the PTX release profiles in cells after incubation with BDP‐SS‐PTX‐NPD and BDP‐PTX‐NPD for varied intervals. Cell viability of BxPC‐3 cells treated with varied concentrations of free PTX, BDP‐OEG, BDP‐PTX‐NPD, and BDP‐SS‐PTX‐NPD for 24 h, (D) without and (E) with light irradiation at 561 nm. (F) CLSM images and (G) the quantitative fluorescence intensity of cells incubated with Rh123 after different treatments. The cell nuclei were stained with DAPI. Data represent the means ± SD, *n* = 3.

The synergistic chemo‐photodynamic therapeutic effect of BDP‐SS‐PTX‐NPD and BDP‐PTX‐NPD was first investigated in human pancreatic cancer BxPC‐3 cells by confocal laser scanning microscopy (CLSM). As shown in Figure 5A, after incubation with the NPDs, cells emitted prominent fluorescence, indicating that they were effectively taken up by the cells. Next, we measured the temporal dynamic level of GSH in cells incubated with the NPDs using Ellman's reagent (5,5′‐dithiobis‐(2‐nitrobenzoic acid) or DTNB), a commonly used commercial GSH probe. As shown in Figure 5B, the intracellular GSH level of cells incubated with BDP‐SS‐PTX‐NPD decreased gradually, reaching a GSH depletion point after 12 h, whereas that of cells incubated with BDP‐PTX‐NPD remained constant under the same experimental conditions. These observations confirmed the high‐efficiency GSH consumption of BDP‐SS‐PTX‐NPD. Meanwhile, we measured the concentration of free PTX in cells over time upon incubation with the NPDs. As shown in Figure 5C, BDP‐SS‐PTX‐NPD released PTX in a time‐dependent fashion, with more drug molecules released with longer incubation time, which, more importantly, is consistent with the GSH consumption rate (Figure 5B). These findings collectively substantiated that the efficient drug release was triggered by GSH consumption in cells.

To examine their therapeutic effects against cancer cells, the in vitro cytotoxicity was measured by the 3‐(4,5‐dimethylthiazol‐2‐yl)‐2,5‐diphenyl tetrazolium bromide (MTT) assay, in which the cell viability of BxPC‐3 cells was calculated after incubation with the NPDs at different concentrations (0–5 µm). Free PTX drug and BDP‐OEG were used as the reference. To evaluate the photodynamic effects, a 561 nm laser was used as the light source to irradiate the PSs with a power of 0.3 W cm^−2^. As shown in Figure 5D, in the absence of light irradiation, BDP‐OEG exhibited negligible toxicity to cells even at high concentrations, implying the high biocompatibility of the PS. Free PTX, BDP‐PTX‐NPD, and BDP‐SS‐PTX‐NPD induced a dose‐dependent inhibitory effect on the proliferation of cancer cells, confirming the chemotherapeutic effects of PTX and the NPDs. In detail, with free PTX as a reference, BDP‐PTX‐NPD generated significantly lower cytotoxicity than PTX, whereas the cytotoxicity of BDP‐SS‐PTX‐NPD was comparable to that of PTX at high concentrations. These results are consistent with the drug release efficiencies of the NPDs (Figure 4A,B). The more efficient release of PTX molecules resulted in the more effective chemotherapeutic effect of BDP‐SS‐PTX‐NPD.

Furthermore, when light irradiation was applied to cells incubated with those treatments for 10 min, BDP‐OEG showed a strong killing effect toward the cells, as shown in Figure 5E, while the cytotoxicity of free PTX remained nearly unchanged. Meanwhile, both BDP‐PTX‐NPD and BDP‐SS‐PTX‐NPD imposed more potent inhibition on cell proliferation compared to their respective non‐irradiation treatments. These findings verified the strong photodynamic effects of the PSs. Notably, little to no living cells were found in the treatment with 5 µm BDP‐SS‐PTX‐NPD, followed by light irradiation, signifying the complete eradication of cancer cells, which should be attributed to therapeutic synergism integrated in the NPD. To validate our hypothesis, we determined the combination index (CI) for quantification of the therapeutic synergism between the PTX and BDP moieties. The CI value (80% inhibition) was calculated to be 0.26, which quantitatively corroborated the strong chemo‐PDT synergism. In addition, we further studied the inhibitory effect of the NPDs on the drug resistance of cancer cells using the fluorescent Rhodamine 123 (Rh123) efflux assay, which is commonly used as an indicator for showcasing the inhibition of P‐glycoprotein (P‐gp) pump. As shown in Figure 5F, cells treated with BDP‐SS‐PTX‐NPD emitted considerably stronger fluorescence than those treated with free PTX and BDP‐PTX‐NPD, which indicated that the P‐gp pump of cells was inhibited by the BDP‐SS‐PTX‐NPD treatment and, as a result, a larger amount of Rh123 was retained in the cells. The fluorescence intensity analysis provided quantitative evidence to further strengthen the higher concentration of Rh123 in those cells (Figure 5G). These findings are consistent with the higher PTX level in cells treated with BDP‐SS‐PTX‐NPD (Figure 5C), resulting in a boosted chemotherapeutic effect. Taken together, the combination of all these results demonstrated that the powerful therapeutic potency of BDP‐SS‐PTX‐NPD results from the prominent synergistic chemotherapeutic‐photodynamic effect.

**FIGURE 6:**
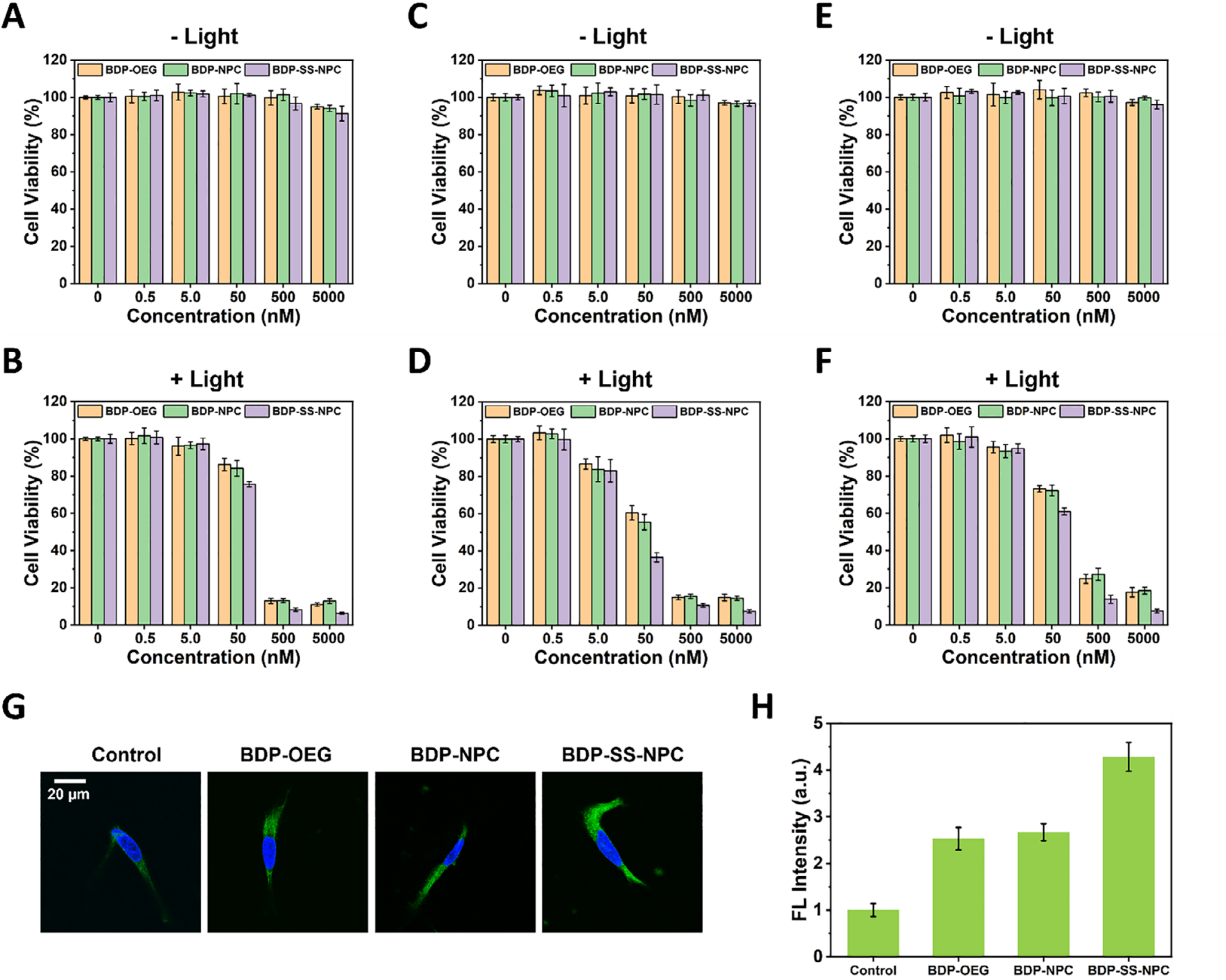
Cell viability of BxPC‐3 (A, B), HeLa (C, D) and SKOV‐3 (E, F) cells treated with varied concentrations of BDP‐OEG, BDP‐NPC, and BDP‐SS‐NPC for 24 h, without (A, C, E), and with (B, D, F) light irradiation at 561 nm. (G) CLSM images and (H) the quantitative fluorescence intensity of cells incubated with DCFH‐DA after different treatments. The cell nuclei were stained with DAPI. Data represent the means ± SD, *n* = 3.

To elucidate the powerful synergistic therapeutic effect of BDP‐SS‐PTX‐NPD, specifically the significantly enhanced cell killing with light irradiation, we hypothesize that the disulfide groups potentiate the photodynamic effect as well, in addition to facilitating the chemotherapeutic effect. To validate the hypothesis, we examined the photodynamic effect of BDP‐OEG, BDP‐NPC, and BDP‐SS‐NPC, excluding the chemotherapeutic effect of PTX in the treatments. Comparison of the data shown in Figure 6A,B revealed that, without light irradiation, the PSs barely affected the viability of cells (Figure 6A), whereas, with light irradiation, PDT occurred when the concentration of the PSs was 50 nm and higher (Figure 6B). More importantly, BDP‐SS‐NPC exerted a stronger photodynamic effect than its non‐SS‐containing counterparts, BDP‐OEG and BDP‐NPC. These outcomes are ascribed to the consumption of GSH during the drug release process (Figure 4D). In addition, the GSH‐consumption‐promoted PDT was further validated in human cervical cancer HeLa cells (Figure 6C,D) and the human ovarian adenocarcinoma SKOV‐3 cells (Figure 6E,F). To further corroborate our hypothesis, we utilized a nonfluorescent, cell‐permeable ROS probe, 2′,7′‐dichlorofluorescein diacetate (DCFH‐DA), which can be rapidly oxidized to a fluorescent compound by intracellular ROS and is thus commonly used as a ROS indicator, to showcase the ROS levels in cells incubated with BDP‐OEG, BDP‐NPC, and BDP‐SS‐NPC upon light irradiation. As shown in Figure 6G, the intracellular ROS level was significantly elevated by the photodynamic effect of the PSs, as evidenced by the increased fluorescence emission. The fluorescence intensity analysis provided solid quantitative evidence to further strengthen the ROS upregulation induced by the PSs (Figure 6H). In detail, BDP‐OEG and BDP‐NPC generated a comparable level of ROS enhancement, which is noticeably lower than that generated by BDP‐SS‐NPC. These observations suggest that the GSH consumption of BDP‐SS‐NPC promoted the enhancement of the intracellular GSH level. As introduced above, cancer cells rely heavily on GSH to neutralize ROS. The GSH consumption reduces the intracellular level of GSH, which consequently diminishes the ability of cancer cells to neutralize the ROS generated by the PSs. As a result, the intracellular ROS accumulates to an unbearable level in a very short period, leading to the apoptosis of cells.

### 2.7 In Vivo Synergistic Chemo‐PDT Tumor Treatment

**FIGURE 7:**
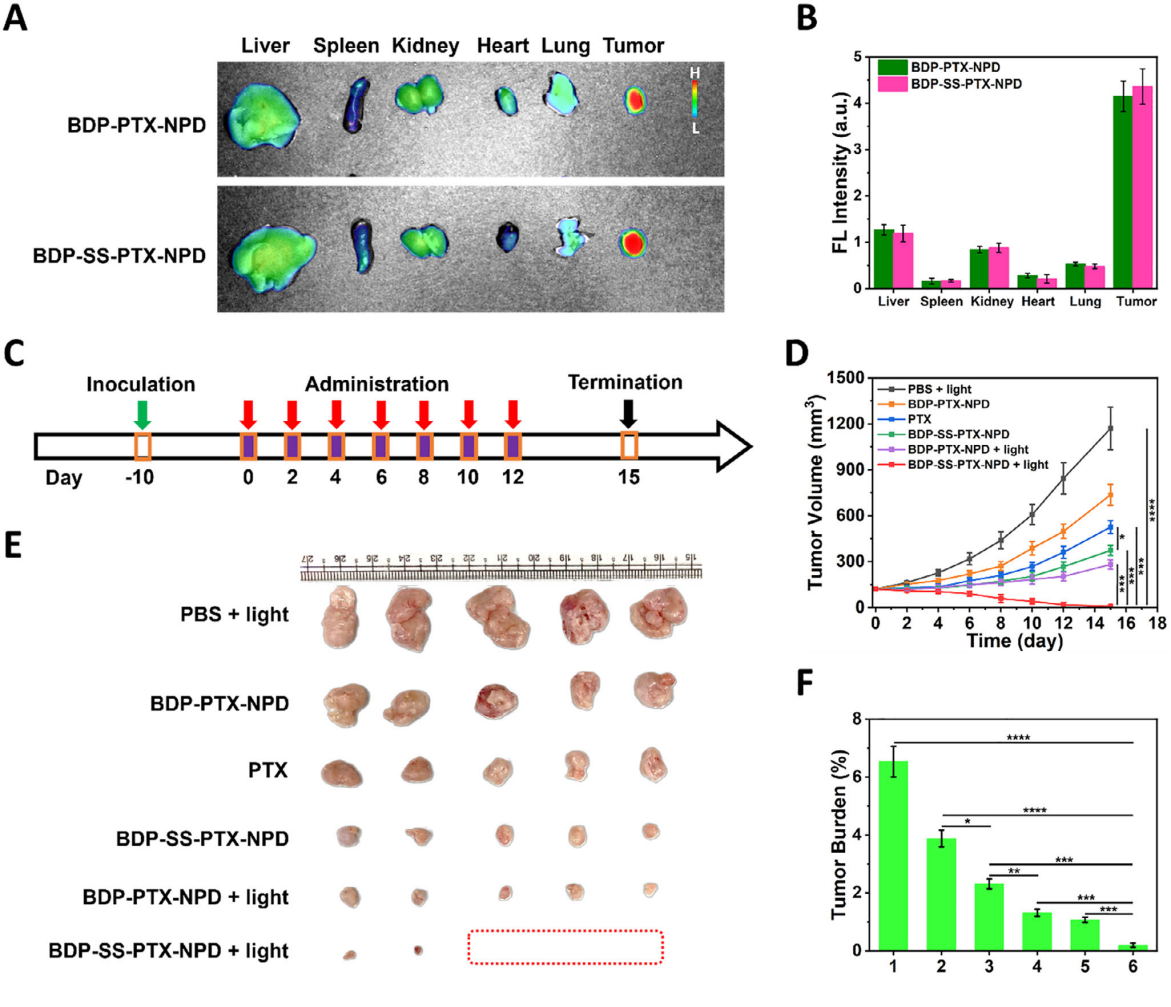
In vivo biodistribution and therapeutic efficacy of BDP‐SS‐PTX‐NPD and BDP‐PTX‐NPD. (A) Fluorescence images and (B) the quantitative fluorescence intensity of major organs and tumors of mice. (C) Treatment timeline for tumor treatment. (D) Tumor growth profiles of mice with varied treatments. (E) Images of tumors harvested from sacrificed mice. (F) Tumor burden analysis of tumors from mice in different treatment groups: (1) PBS + light, (2) BDP‐PTX‐NPD, (3) free PTX, (4) BDP‐SS‐PTX‐NPD, (5) BDP‐PTX‐NPD + light, and (6) BDP‐SS‐PTX‐NPD + light. Data represent the means ± SD, *n* = 5. ^*^ *p* < 0.05; ^**^ *p* < 0.01; ^***^ *p* < 0.001; and ^****^ *p* < 0.0001.

Encouraged by the exceptional cancer cell‐killing potency of the BDP‐SS‐PTX‐NPD in the in vitro experiments, we continued to explore its powerful therapeutic effect to treat tumors in a xenograft tumor model of BxPC‐3 cancer in mice. To assess the self‐delivering efficiency of the NPDs into tumor tissues, we analyzed the in vivo biodistribution of BDP‐PTX‐NPD and BDP‐SS‐PTX‐NPD in tumor‐bearing mice using a Lago X animal imaging system. Mice were intravenously administered the NPDs dispersed in sterile PBS at a dose of 1 mg/kg. The animals were euthanized 24 h post‐injection, and major organs (heart, liver, spleen, lungs, and kidneys), along with tumor tissues, were harvested for ex vivo imaging to evaluate the biodistribution of the NPDs. As shown in Figure 7A, strong fluorescence signals were observed in the tumor tissues. Quantitative analysis data further revealed that the fluorescence intensity in tumors was significantly higher than that in other organs (Figure 7B), indicating preferential accumulation and prolonged retention of the self‐delivering NPDs within tumor sites.

Moving forward, we next evaluated the in vivo therapeutic efficacy of the synergistic chemo‐PDT NPDs against pancreatic tumors with and without light irradiation at 561 nm. As outlined in Figure 7C, the experiment encompassed tumor inoculation, treatment administration, and final assessment. BxPC‐3 tumor‐bearing mice were randomly assigned to six groups and administered with different treatments every other day, including sterile PBS + light irradiation (control group), free PTX drug, BDP‐PTX‐NPD, BDP‐SS‐PTX‐NPD, BDP‐PTX‐NPD + light irradiation, and BDP‐SS‐PTX‐NPD + light irradiation. The light irradiation was applied to the tumor sites with a power of 0.5 W cm^−2^ for 5 min. Tumor growth profiles following the different treatments are presented in Figure 7D. In the control group, tumors exhibited rapid and unchecked growth, reaching approximately a 10‐fold increase in relative tumor volume after two weeks. Treatment with free PTX moderately suppressed tumor progression, resulting in an approximately fourfold increase in tumor volume by the end of the study. Among all the treatments, BDP‐PTX‐NPD in the absence of light irradiation exhibited the lowest efficiency in inhibiting tumor growth. In contrast, the GSH‐consuming BDP‐SS‐PTX‐NPD generated a more potent chemotherapeutic effect against the tumor, due to the GSH‐triggered traceless release of PTX molecules. More importantly, BDP‐SS‐PTX‐NPD demonstrated significantly higher antitumor efficiency than free PTX, ascribed to the EPR effect of the nanomedicine and the inhibition of drug resistance resulting from GSH consumption. Furthermore, with the application of light irradiation, both BDP‐PTX‐NPD and BDP‐SS‐PTX‐NPD exerted substantially elevated inhibitory effects on tumor growth, as a result of the synergistic chemo‐PDT effect. Exceptionally, the treatment of BDP‐SS‐PTX‐NPD + light irradiation produced a pronounced antitumor efficacy, initially shrinking tumor burdens and finally resulting in the disappearance of tumors, attributed to the GSH‐consumption‐promoted PDT. These findings showcased the powerful therapeutic potency of the synergistic GSH‐consuming chemo‐PDT in treating tumors in vivo.

**FIGURE 8:**
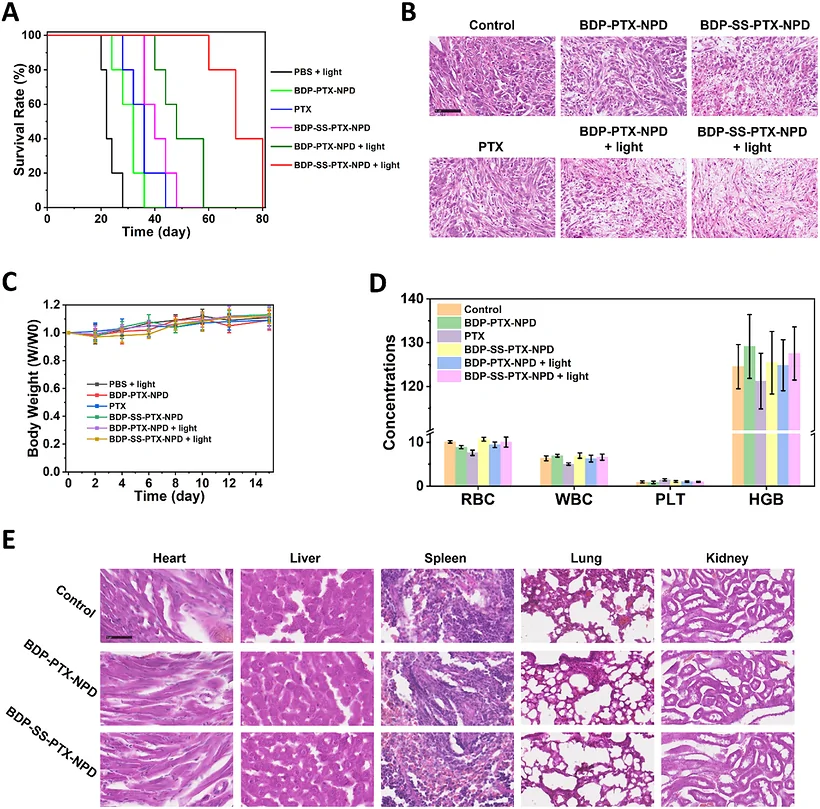
(A) Survival curves of tumor‐bearing mice following various treatments. (B) H&E staining images of the collected tumors of mice after different treatments. (C) Changes in the body weight of mice over time. (D) Hematological analysis of mice after different treatments. RBC, M/µL; WBC, K/µL; PLT, M/µL; HGB, g/L. (E) H&E staining images of the collected main organs of mice after different treatments. Data represent the means ± SD, *n* = 5.

At the end of the experiment, the tumor tissues of the mice were excised for further analysis. As displayed in Figure 7E, large tumor solids were collected from the mice in the control group, whereas those from the experimental groups had smaller sizes to varying degrees, which is in good agreement with the observations in the tumor growth, indicating the effectiveness of the treatments. Notably, among the 5 mice treated with BDP‐SS‐PTX‐NPD + light irradiation, only a tiny piece of tumor was found in 2 mice, and no visible tumor solids were found in the other 3 mice, which signifies the eradication of tumors in those mice. The results were further corroborated by the quantitative analysis of tumor weight (Figure 7F). Moreover, the mice treated with the synergistic PDT treatments exhibited considerably longer survival times than those in the other treatment groups (Figure 8A). Further, histological examination of excised tumor tissues from sacrificed mice using hematoxylin and eosin (H&E) staining demonstrated that tumors from mice in the experimental groups exhibited pronounced cell apoptosis and markedly reduced cellular proliferation compared with those from the control group (Figure 8B). Especially, the tissue textures of tumors in mice administered with synergistic chemo‐PDT treatments, BDP‐PTX‐NPD/BDP‐SS‐PTX‐NPD + light irradiation, were substantially destroyed, underscoring the powerful cancer cell‐killing potency of the therapeutics. In addition, no appreciable loss in body weight was observed across all treatment groups throughout the study period (Figure 8C). Hematological analysis showed that routine blood test parameters, including red blood cells (RBC), white blood cells (WBC), platelets (PLT), and hemoglobin (HGB), of mice in the experimental groups were at the same levels as those in the control group (Figure 8D), suggesting that the treatments were well tolerated and did not induce significant systemic toxicity. Consistent with that, histological analysis of major organs, including the heart, liver, spleen, lungs, and kidneys, revealed no evident pathological abnormalities, such as inflammation, necrosis, or structural disruption, in any of the examined tissues (Figure 8E), indicating that normal physiological functions and overall health status were largely unaffected. These observations demonstrate a favorable biosafety profile, supporting the minimal off‐target toxicity of the treatments.

## 3 Conclusion

We developed a chemo‐photodynamic prodrug, BDP‐SS‐PTX, that can self‐assemble into a carrier‐free, stimuli‐responsive nanoprodrug, BDP‐SS‐PTX‐NPD, in aqueous solution, exploring a GSH‐consumption‐promoted synergistic chemo‐PDT effect to achieve boosted anticancer therapy with a favorable biosafety profile. The prodrug molecules consist of PTX and BODIPY moieties conjugated through a GSH‐responsive self‐immolating drug linker, enabling traceless release of PTX molecules at the consumption of intracellular GSH, and consequently reduced drug resistance and disabled ROS neutralization of cancer cells. The NPD has a diameter of approximately 65 nm and an ultrahigh drug loading content (>70%). In comparison with its non‐GSH‐consuming counterpart, BDP‐PTX‐NPD, BDP‐SS‐PTX‐NPD exhibited efficient PTX release in response to the presence of GSH and significantly higher cancer‐killing efficiencies in both in vitro and in vivo evaluations. Due to the inherent fluorescence emission of the BODIPY moiety, the in vivo biodistribution studies revealed enhanced accumulation and retention of the NPDs in tumor tissues, benefiting from their optimal nanoscale dimensions. Remarkably, the GSH‐consuming theranostic nanoprodrug demonstrated superior therapeutic efficacy compared with its non‐GSH‐consuming counterpart. This enhanced performance is attributed to several integrated advantages, including GSH‐triggered traceless release of PTX, attenuation of drug resistance, and impaired ROS detoxification caused by intracellular GSH consumption, ultimately leading to complete tumor eradication. Also, the treatment induced no significant systemic toxicity and adverse side effects, underscoring its additional therapeutic advantage. Altogether, the results highlight the robust antitumor activities of the synergistic chemo‐PDT approaches while maintaining a favorable biocompatibility profile, and in particular, the strong potential of the GSH‐consumption‐promoted BDP‐SS‐PTX‐NPD as a safe and effective treatment strategy, warranting further investigation toward clinical translation.

## Author Contributions

**Binglin Sui**: conceptualization, methodology, formal analysis, investigation, funding acquisition, project administration, Writing – review and editing, Writing – original draft. **Hansheng Huang**: software, data curation, investigation, validation, methodology, formal analysis.

## Conflicts of Interest

The authors declare no conflicts of interest.

## Supporting information




**Supporting File**: advs77484‐sup‐0001‐SuppMat.pdf.

## Data Availability

The data that support the findings of this study are available from the corresponding author upon reasonable request.
