# Supplementary material for: Carrier‐Free Stimulus‐Responsive Theranostic Nanoprodrug Enabling GSH‐Consumption‐Promoted Chemo‐Photodynamic Cancer Therapy
Source: Adv Sci (Weinh). 2026 Sep 13:e77484. Online ahead of print. doi: 10.1002/advs.77484 (PMC13572825; doi:10.1002/advs.77484)
Supplement: Supplementary file 1 [file ADVS-9999-e77484-s001.pdf]

Supporting Information

Carrier-free stimulus-responsive theranostic nanoprodrug enabling GSH-consumption-promoted chemo-photodynamic cancer therapy

Hansheng Huang, Binglin Sui*

Department of Chemistry, University of North Dakota, Grand Forks, North Dakota 58202, United States

*Corresponding author. E-mail address: binglin.sui@und.edu

Materials and Methods

Materials

Hexaethylene glycol, *p*-toluenesulfonyl chloride (TsCl), triethylamine (TEA), $NaHCO_3$, 4-hydroxybenzaldehyde, K_2CO_3, KI, trifluoroacetic acid (TFA), 2,3-dichloro-5,6-dicyano-1,4-benzoquinone (DDQ), boron trifluoride diethyl ether complex (BTF), pyridine, 4-nitrophenyl chloroformate (NPC), 2-hydroxyethyl disulfide, paraformaldehyde (PFA), glutathione (GSH), phosphate-buffered saline (PBS), 3-(4,5-dimethylthiazol-2-yl)-2,5-diphenyltetrazolium bromide (MTT), and silica gel (spherical, 100 μm) were purchased from Thermo Fisher Scientific, Inc. (Waltham, MA, USA). Cremophor EL® was purchased from EMD Millipore Corp. (Billerica, MA, USA). Paclitaxel (PTX) was purchased from LC Laboratories (Woburn, MA, USA). Gibco Dulbecco's modified Eagle's medium (DMEM), Roswell Park Memorial Institute (RPMI) 1640 medium (Gibco™), fetal bovine serum (FBS), penicillin-streptomycin (PS), trypsin-EDTA, and 4',6-diamidino-2-phenylindole dihydrochloride (DAPI) were purchased from Thermo Fisher Scientific, Inc. (Waltham, MA, USA). Deuterated chloroform was purchased from Cambridge Isotope Laboratories, Inc. (Andover, MA, USA). All of the solvents used in this research were purchased from Thermo Fisher Scientific, Inc. (Waltham, MA, USA).

6-Methyl-4*H*-thieno[3,2-*b*]pyrrole and compound **3** were synthesized following the respective procedures described in the literature.[1,2]

Synthesis of compound 1

TsCl (191 mg, 1.0 mmol) in 5 mL of DCM was added dropwise to a stirred solution of OEG6 (565 mg, 2.0 mmol) and TEA (420 μL, 3.0 mmol) in 10 mL of DCM. The reaction mixture was stirred for 8 h at room temperature. Then the reaction mixture was washed with saturated $NaHCO_3$ solution. The organic phase was condensed, and the crude product was purified by flash column chromatography using silica gel as the stationary phase and ethyl acetate/hexane as the mobile phase. The final product was obtained as a colorless oil (323 mg, 74%). ^1H NMR (400 MHz, $CDCl_3$): δ (ppm) 7.80 (d, J = 8.0, 2H), 7.34 (d, J = 8.0, 2H), 4.16 (t, J = 4.6, 2H), 3.72-3.59 (m, 22H), 2.45 (s, 3H). ^{13}C NMR (101 MHz, $CDCl_3$): δ (ppm) 144.81, 133.03, 129.83, 127.99, 72.49, 70.74, 70.70, 70.61, 70.55, 70.50, 70.26, 69.27, 68.67, 61.69, 53.44, 21.63.

Synthesis of compound 2

A mixture of 4-hydroxybenzaldehyde (244 mg, 2.0 mmol), compound **1** (437 mg, 1.0 mmol), KI (166 mg, 1.0 mmol), and K_2CO_3 (276 mg, 2.0 mmol) in DMF (2 mL) was heated to 120 °C for 3 h. After evaporation under reduced pressure, DCM was added to the mixture. The mixture was filtered, and the solution was washed with DI water. After condensation, the crude product was purified by column chromatography using ethyl acetate/methanol as an eluent to give the product as a colorless oil (683 mg, 88%). ^{1}H NMR (400 MHz, $CDCl_3$): δ (ppm) 9.88 (s, 1H), 7.83 (d, *J* = 7.7, 2H), 7.03 (d, *J* = 7.7, 2H), 4.22 (t, *J* = 4.3, 2H), 3.89 (t, *J* = 4.3, 2H), 3.74-3.59 (m, 20H). ^{13}C NMR (101 MHz, $CDCl_3$): δ (ppm) 190.83, 163.87, 131.96, 130.06, 114.90, 72.55, 70.88, 70.63, 70.60, 70.55, 70.52, 70.29, 69.48, 67.78, 61.73, 53.43.

Synthesis of BDP-OEG

A solution of compound **2** (386 mg, 1.0 mmol), 6-methyl-4*H*-thieno[3,2-*b*]pyrrole (302 mg, 2.2 mmol), and TFA (2 drops) in 20 mL of anhydrous DCM was stirred at room temperature for 4 h. Then, DDQ (270 mg, 1.2 mmol) was added over 20 min. The reaction mixture was stirred for another 1 h. After the addition of 2.0 mL of TEA, 2.5 mL of BTF was added dropwise to the mixture. The mixture was kept stirring at room temperature for 16 h, then filtered through a Celite pad. The crude product was purified by silica gel column chromatography using a mixed eluent of ethyl acetate/methanol. The product was obtained as a pink solid (156 mg, 23%). ^{1}H NMR (400 MHz, $CDCl_3$): δ (ppm) 7.60 (t, *J* = 2.1, 2H), 7.25 (d, *J* = 8.3, 2H), 7.15 (d, *J* = 2.7, 2H), 7.09 (d, *J* = 8.3, 2H), 4.22 (s, 2H), 3.93 (s, 2H), 3.77-3.61 (m, 21H), 1.63 (s, 6H). ^{13}C NMR (101 MHz, $CDCl_3$): δ (ppm) 159.88, 156.94, 145.93, 140.16, 137.96, 134.00, 133.65, 129.50, 126.13, 115.49, 114.17, 72.49, 70.84, 70.61, 70.56, 70.53, 70.50, 70.48, 70.21, 69.70, 67.58, 61.67, 14.28.

Synthesis of BDP-NPC

NPC (302 mg, 1.5 mmol) was slowly added to a stirring solution of BDP-OEG (344 mg, 0.5 mmol) and pyridine (160 µL, 2 mmol) in 10 mL of anhydrous DCM. The reaction was stirred at room temperature for 16 h. After condensation, the crude product was purified by flash column chromatography using silica gel as the stationary phase and a mixture of ethyl acetate and acetone as the mobile phase. The final product was obtained as a pink solid (381 mg, 89%). ^{1}H NMR (400 MHz, $CDCl_3$): δ (ppm) 8.27 (d, *J* = 8.2, 2H), 7.60 (d, *J* = 4.6, 2H), 7.38 (d, *J* = 8.2, 2H), 7.26 (s, 2H), 7.15 (d, *J* = 4.7, 2H), 7.09 (d, *J* = 8.2, 2H), 4.43 (s, 2H), 4.22 (s, 2H), 3.93 (s, 2H), 3.80-3.67 (m, 18H), 1.63 (s, 6H). ^{13}C NMR (101 MHz,

$CDCl_3$): δ (ppm) 159.86, 156.94, 155.50, 152.50, 145.89, 145.41, 140.16, 137.96, 133.97, 133.64, 129.52, 126.18, 125.32, 121.77, 115.48, 114.19, 70.93, 70.72, 70.66, 70.65, 70.61, 69.72, 68.64, 68.29, 67.60, 14.29.

Synthesis of BDP-PTX

A solution of BDP-NPC (85 mg, 0.1 mmol), PTX (170 mg, 0.2 mmol), and TEA (70 µL, 0.5 mmol) in 2 mL of anhydrous DCM was stirred at room temperature for 20 h. The mixture was condensed under reduced pressure. The crude product was purified by flash column chromatography using silica gel as the stationary phase and a mixture of ethyl acetate/methanol as the mobile phase. The final product was obtained as a pink solid (113 mg, 72%). ^{1}H NMR (400 MHz, $CDCl_3$): δ (ppm) 8.14 (d, *J* = 7.2, 2H), 7.75 (d, *J* = 7.2, 2H), 7.60 (d, *J* = 3.2, 3H), 7.53-7.47 (m, 3H), 7.44-7.31 (m, 7H), 7.24 (s, 1H), 7.15 (d, *J* = 4.7, 2H), 7.08 (d, *J* = 7.2, 2H), 6.96 (d, *J* = 8.4, 1H), 6.32-6.25 (m, 2H), 5.98 (d, *J* = 6.6, 1H), 5.69 (d, *J* = 6.0, 1H), 5.42 (s, 1H), 4.97 (d, *J* = 9.2, 1H), 4.47-4.19 (m, 7H), 3.91 (s, 2H), 3.81 (d, *J* = 6.0, 1H), 3.76-3.62 (m, 19H), 3.48 (s, 1H), 2.62-2.36 (m, 6H), 2.23-2.16 (m, 4H), 1.92-1.85 (m, 4H), 1.68 (s, 3H), 1.63 (s, 6H), 1.24 (s, 3H), 1.14 (s, 3H). ^{13}C NMR (101 MHz, $CDCl_3$): δ (ppm) 203.82, 171.28, 169.89, 167.87, 167.09, 159.86, 156.95, 154.20, 145.87, 142.74, 140.15, 137.96, 136.78, 133.95, 133.71, 133.64, 133.51, 132.81, 132.03, 130.25, 129.51, 129.16, 129.11, 128.77, 128.69, 128.51, 127.21, 126.61, 126.18, 115.47, 114.20, 84.44, 81.08, 79.23, 76.70, 76.46, 75.59, 75.10, 72.16, 72.03, 70.92, 70.66, 70.63, 70.60, 70.57, 69.72, 68.68, 68.12, 67.59, 58.54, 52.70, 45.56, 43.20, 35.57, 26.85, 22.72, 22.18, 20.84, 14.82, 14.29, 9.61.

Synthesis of BDP-SS-NPC

A solution of BDP-OEG (344 mg, 0.5 mmol), compound **3** (727 mg, 1.5 mmol), and TEA (280 µL, 2 mmol) in 5 mL of anhydrous DCM was stirred at room temperature for 20 h. After condensation, the crude product was purified by flash column chromatography using silica gel as the stationary phase and a mixture of ethyl acetate and acetone as the mobile phase. The final product was obtained as a pink solid (274 mg, 53%). ^{1}H NMR (400 MHz, $CDCl_3$): δ (ppm) 8.28 (d, *J* = 9.0, 2H), 7.60 (d, *J* = 5.2, 2H), 7.39 (d, *J* = 9.0, 2H), 7.25 (s, 4H), 7.15 (d, *J* = 5.2, 2H), 7.09 (d, *J* = 8.3, 2H), 4.54 (t, *J* = 6.2, 2H), 4.40 (t, *J* = 6.5, 2H), 4.29 (t, *J* = 4.3, 2H), 4.22 (t, *J* = 4.2, 2H), 3.93 (t, *J* = 4.4, 2H), 3.78-3.65 (m, 18H), 3.04 (t, *J* = 6.2, 2H), 2.98 (t, *J* = 6.5, 2H), 1.62 (s, 6H). ^{13}C NMR (101 MHz, $CDCl_3$): δ (ppm) 159.88, 156.94, 155.40,

154.90, 152.31, 145.91, 145.50, 140.14, 137.96, 133.98, 133.65, 129.52, 126.17, 125.35, 121.81, 115.48, 114.20, 70.94, 70.66, 70.60, 69.73, 68.85, 67.61, 67.25, 66.78, 65.59, 37.03, 36.78, 14.30.

Synthesis of BDP-SS-PTX

A solution of BDP-SS-NPC (52 mg, 0.05 mmol), PTX (85 mg, 0.1 mmol), and TEA (30 µL, 0.2 mmol) in 2 mL of anhydrous DCM was stirred at room temperature for 20 h. The mixture was condensed under reduced pressure. The crude product was purified by flash column chromatography using silica gel as the stationary phase and a mixture of ethyl acetate/methanol as the mobile phase. The final product was obtained as a pink solid (67 mg, 77%). ^{1}H NMR (400 MHz, $CDCl_3$): δ (ppm) 8.14 (d, J = 7.7, 2H), 7.75 (d, J = 7.7, 2H), 7.61 (d, J = 5.2, 3H), 7.53-7.47 (m, 3H), 7.44-7.35 (m, 7H), 7.24 (s, 1H), 7.15 (d, J = 5.2, 2H), 7.09 (d, J = 8.0, 2H), 7.01 (d, J = 9.2, 1H), 6.30-6.25 (m, 2H), 6.00 (d, J = 9.2, 1H), 5.69 (d, J = 6.8, 1H), 5.43 (s, 1H), 4.97 (d, J = 9.2, 1H), 4.44-4.21 (m, 11H), 3.92 (s, 2H), 3.82-3.63 (m, 19H), 2.96-2.88 (m, 4H), 2.59-2.37 (m, 6H), 2.23-2.17 (m, 4H), 1.93-1.85 (m, 4H), 1.76 (s, 1H), 1.68 (s, 3H), 1.63 (s, 6H), 1.27 (s, 3H), 1.14 (s, 3H). ^{13}C NMR (101 MHz, $CDCl_3$): δ (ppm) 203.82, 171.26, 169.88, 167.82, 167.16, 167.05, 159.88, 156.94, 154.90, 153.94, 145.92, 142.65, 140.16, 137.96, 136.69, 133.99, 133.70, 133.65, 133.54, 132.86, 132.02, 130.24, 129.51, 129.18, 129.14, 128.76, 128.68, 128.55, 127.21, 126.63, 126.15, 115.48, 114.19, 84.44, 81.08, 79.17, 76.71, 76.45, 75.59, 75.10, 72.14, 72.11, 70.92, 70.63, 70.58, 69.71, 68.81, 67.60, 67.22, 66.69, 65.58, 58.53, 52.70, 45.57, 43.20, 36.98, 36.71, 35.54, 26.83, 22.73, 22.15, 20.83, 14.84, 14.29, 9.60.

Photophysical properties

UV-Vis absorption spectra and fluorescence emission spectra were recorded using a PerkinElmer Lambda 1050 UV/Vis/NIR spectrophotometer and a Shimadzu RF-6000 spectrophotometer, respectively. The spectroscopic analysis of the BODIPY materials was conducted in 10 mm path-length quartz cuvettes at room temperature. Each measurement was conducted with an optical density limited to 0.12 or lower at the excitation wavelength to prevent reabsorption.

Fluorescence emission quantum yields

The fluorescence emission quantum yields (Φ_{FL}) of the BODIPY materials were determined by a standard relative method using Rhodamine 6G (Φ_{FL} = 0.94 in ethanol) as a reference standard, according to the equation below.[3]

$$\Phi_{FL} = \Phi_R \frac{I}{I_R} \frac{OD_R}{OD} \frac{n^2}{n_R^2} \frac{RP_R}{RP}$$

where Φ_R is the fluorescence emission quantum yield of the reference standard, *I* is the integrated area under the emission signal, *OD* is the optical density at the excitation wavelength, *n* is the refractive index of the used solvent, and *RP* is the relative power of the light source at the excitation wavelength of the spectrofluorometer.

Singlet oxygen quantum yields

The singlet oxygen quantum yields (Φ_{SO}) of the BODIPY photosensitizers were determined using a comparative spectrophotometric method. 1,3-Diphenylisobenzofuran (DPBF) was used as the $^1O_2$ quencher and rose bengal (RB, Φ_{SO} = 0.54 in ACN) as the reference standard. Mixed solutions of DPBF (abs ~ 1.00) and a photosensitizer (abs ~ 0.2) in air-saturated ACN were kept in the dark. Upon continuous light irradiation, the absorbance of DPBF at 414 nm was measured every 2 seconds to plot the absorbance decay rate of the photosensitizing course, absorbance versus irradiation time. The Φ_{SO} values of the BODIPY photosensitizers were calculated according to the equation below.

$$\Phi_{SO} = \Phi_R \frac{S}{S_R} \frac{F_R}{F} \frac{PF_R}{PF}$$

where Φ_R is the singlet oxygen quantum yield of the reference standard, *S* is the slope of the photodegradation curve of DPBF versus the irradiation time, *F* is the absorption correction factor for the difference in optical density (OD), which is given as $F = 1\text{-}10^{-OD}$, and PF is the absorbed photonic flux (μEinstein dm^{-3} s^{-1}).

Fabrication of carrier-free nanoprodrugs

Nanoprodrugs BDP-PTX-NPD and BDP-SS-PTX-NPD were fabricated following an established nano-assembling procedure, as detailed in our earlier work.[2] The BDP-PTX or BDP-SS-PTX prodrug (10 mg) was first dissolved in 10 mL of acetone and subsequently introduced into 100 mL of deionized (DI) water under vigorous stirring. Upon dispersion in the aqueous phase, the amphiphilic prodrug molecules spontaneously self-assembled into nanoscale structures to form nanoprodrug particles (NPDs). The acetone in the resulting suspension was then removed under reduced pressure at room temperature. The prepared NPDs were stored at 4 °C for subsequent experiments and characterization.

Particle size and surface charge of the NPDs

The hydrodynamic particle size, polydispersity index (PDI), and surface charge of the NPDs were characterized by dynamic light scattering (DLS) using a Zetasizer (Nano ZS, Malvern Instruments Ltd., Malvern, UK). In addition, the morphology and structural features of the BDP-PTX NPDs were examined using a Hitachi HT7800 transmission electron microscopy (TEM, Hitachi High-Technologies Corporation, Tokyo, Japan). The drug-loading content was calculated according to the following equation:

$$\text{Drug loading content (\%)} = \frac{\text{weight of the drug moieties in NPDs}}{\text{weight of the NPDs}} \times 100\%$$

Colloidal and serum stability

The colloidal stability of BDP-PTX-NPD and BDP-SS-PTX-NPD was evaluated by dynamic light scattering (DLS) analysis. Specifically, the hydrodynamic diameter of the nanoprodrugs dispersed in DI water was continuously monitored over a one-week period to assess their stability during storage. To further investigate their serum stability under physiologically relevant conditions, the nanoprodrugs were incubated in phosphate-buffered saline (PBS, pH 7.4) as well as PBS containing 10% fetal bovine serum (FBS, pH 7.4) at 37 °C. These conditions were used to mimic different biological environments encountered during systemic circulation. At predetermined time intervals, the hydrodynamic size of the nanoparticles was measured by DLS to evaluate potential aggregation, structural instability, or protein-induced changes in particle size.

Drug release profiles

The release behaviors of PTX from BDP-PTX-NPD and BDP-SS-PTX-NPD were investigated in various PBS (pH 7.4) solutions containing 20% (v/v) ethanol, which served as the release media, at 37 °C. Different release conditions were employed to simulate distinct physiological environments, including PBS alone, PBS supplemented with 10% FBS, and PBS containing varied concentrations (0.2, 2.0, 10 mM) of glutathione (GSH). For each experiment, BDP-PTX-NPD or BDP-SS-PTX-NPD containing an equivalent of 200 nmol of PTX was loaded into dialysis tubing with a molecular weight cutoff (MWCO) of 6-8 kDa. The sealed dialysis bags were then immersed in pre-equilibrated release media and incubated at 37 °C under continuous conditions. At predetermined time intervals, aliquots of the external release media were collected and analyzed by high-performance liquid chromatography (HPLC) to quantify the amount of released PTX. In addition, the chemical species present in the final release media were further

characterized by high-resolution mass spectrometry (HRMS) to investigate the degradation products and release mechanisms of the nanoprodrugs.

Cell culture

Human pancreatic cancer BxPC-3 cells were cultured in RPMI 1640 cell culture medium, supplemented with 10% FBS, 100 units/mL penicillin, and 100 μg/mL streptomycin in 100 mm cell culture dishes at 37 °C under a humidified atmosphere of 5% CO_2. Human cervical cancer HeLa cells and ovarian adenocarcinoma SKOV-3 cells were cultured in DMEM cell culture medium, supplemented with 10% FBS, 100 units/mL penicillin, and 100 μg/mL streptomycin in 100 mm cell culture dishes at 37 °C under a humidified atmosphere of 5% CO_2. Cells were subcultured when the cell confluence reached approximately 70-80%.

Confocal fluorescence imaging

Cellular fluorescence imaging was carried out using confocal laser scanning microscopy (CLSM). Cells were seeded in 35 mm glass-bottom Petri dishes at a density of 2×10^5 cells per dish and cultured for 24 h at 37 °C in a humidified atmosphere containing 5% CO_2. After washing with PBS (pH 7.4), the cells were incubated with BDP-PTX-NPD or BDP-SS-PTX-NPD at a concentration of 1 μM for 3 h. Cells maintained in fresh medium without BDP-PTX-NPD or BDP-SS-PTX-NPD treatment were used as the control group. Following incubation, all samples were washed three times with PBS and fixed with 4% paraformaldehyde in PBS for 10 min at room temperature. The fixative was then removed, and the cells were rinsed three additional times with PBS. Subsequently, the nuclei were stained with DAPI (1 μg/mL) for 10 min, followed by three further washes with PBS. Confocal fluorescence images were then acquired using an Olympus FV3000 Laser Scanning Confocal Microscope.

Intracellular GSH measurement

To measure the temporal GSH level in cells, cells were seeded in 6-well plates at a density of 1,000,000 cells per well and cultured for 24 h at 37 °C in a humidified incubator with 5% CO_2. After washing with PBS (pH 7.4), cells were incubated with the NPDs at a concentration equivalent to 20 μM PTX. At predetermined time intervals, the cell culture medium was removed, and the cells were treated with Ellman’s reagent (5,5′-dithiobis-(2-nitrobenzoic acid) or DTNB) following the standard Ellman’s assay

protocol. The optical absorbance at 412 nm was measured using a microplate reader (BioTek Synergy H1 multimode microplate reader).

Intracellular drug release

To measure the intracellular drug release, cells were seeded in 6-well plates at a density of 1,000,000 cells per well and cultured for 24 h at 37 °C in a humidified incubator with 5% CO_2. After washing with PBS (pH 7.4), cells were incubated with the NPDs at a concentration equivalent to 20 μM PTX. At predetermined time intervals, the cell culture medium was removed, and the cells were treated with RIPA lysis buffer. The cell lysate mixtures were shaken gently at room temperature for 15 min. The final cell lysates, combined with the cell culture medium, were extracted with dichloromethane. The collected extract was combined and analyzed by HPLC to measure the released PTX in the mixture.

Cell viability assay

The cytotoxicity of BDP-PTX-NPD and BDP-SS-PTX-NPD was studied by the 3-(4,5-dimethylthiazole-2-yl)-2,5-diphenyltetrazolium bromide (MTT) assay in BxPC-3, HeLa, and SKOV-3 cells. Cells were seeded at a density of 5,000 cells per well into 96-well plates and cultured at 37 °C in a humidified atmosphere containing 5% CO_2 for 24 h prior to use. For the experimental groups, cells were incubated in fresh cell culture medium supplemented with free PTX, the BODIPY photosensitizers, or the NPDs at various concentrations for 24 h. Control cells were cultured under identical conditions in fresh medium without the addition of PTX, the BODIPY photosensitizers, or the NPDs. For cells treated with additional light irradiation, cells were incubated in fresh cell culture medium supplemented with the BODIPY photosensitizers or the NPDs for the initial 2 h, followed by light irradiation at 561 nm with a power of 0.3 W cm^{-2} for 10 min, and further incubated for 22 h. Following incubation, the cell culture medium was replaced with fresh medium containing the MTT reagent at a final concentration of 1 mg/mL, and the cells were incubated for an additional 4 h. After removing the medium, 100 μL of DMSO was added to each well to dissolve the formed formazan crystals during the MTT treatment. Then, the absorbance at 570 nm was measured using a microplate reader (BioTek Synergy H1 multimode microplate reader).

Combination index calculation

To verify whether the combination of PTX and BDP moieties can attain chemo-PDT therapy in a synergistic manner, the combination index (CI) was determined based on the concentrations of each

treatment at 80% inhibition of cell growth obtained by the MTT assay. The Chou-Talalay method was used to calculate the CI value, according to the equation below.[4]

$$\mathrm{CI} = \frac{C_{\mathrm{BDP-SS-PTX-NPD}}}{C_{\mathrm{PTX}}} + \frac{C_{\mathrm{BDP-SS-PTX-NPD}}}{C_{\mathrm{BDP}}}$$

Where CI < 1, = 1, and > 1 indicate synergism, additive effect, and antagonism, respectively; $C_{\text{BDP-SS-PTX-NPD}}$ is the concentration of BDP-SS-PTX-NPD; C_{PTX} is the concentration of free PTX drug; and C_{BDP} is the concentration of BDP. The smaller the CI value, the stronger the synergistic effect.

Intracellular ROS measurement

Intracellular ROS levels of cells were measured by CLSM, using 2′,7′-dichlorofluorescein diacetate (DCFH-DA) as the fluorescent indicator. Cells were seeded at a density of 150,000 cells per dish in 35-mm glass-bottom Petri dishes and cultured for 24 h at 37 °C in a humidified incubator with 5% CO_2. After washing with PBS (pH 7.4), cells were incubated with the BDP derivatives at a concentration of 5 μM for 6 h, followed by light irradiation at 561 nm with a power of 0.3 W cm^{-2} for 10 min. Cells maintained in fresh medium without treatment served as the control. After washing with PBS, the cells were treated with DCFH-DA for 30 min. The cell nuclei were stained with DAPI for 10 min. Following the final washing with PBS, the intracellular fluorescence emission was measured by the Olympus FV3000 Laser Scanning Confocal Microscope.

Intracellular drug efflux

Intracellular drug efflux of cells was measured by CLSM, using the commercially available Rhodamine 123 (Rh123) as the fluorescent indicator. Cells were seeded at a density of 150,000 cells per dish in 35-mm glass-bottom Petri dishes and cultured for 24 h at 37 °C in a humidified incubator with 5% CO_2. After washing with PBS (pH 7.4), cells were incubated with free PTX or NPDs at a concentration of 5 μM for 6 h, followed by light irradiation at 561 nm with a power of 0.3 W cm^{-2} for 10 min. Cells maintained in fresh medium without treatment served as the control. After incubation for 6 h, the cells were treated with Rh123 for 30 min. After washing with PBS, the cells were incubated in fresh cell culture medium for another 6 h, and then the cell nuclei were stained with DAPI for 10 min. Following the final washing with PBS, the intracellular fluorescence emission was measured by the Olympus FV3000 Laser Scanning Confocal Microscope.

Animal model

All animal experiments were conducted following NIH regulations and approved by the Institutional Animal Care and Use Committee (IACUC) of the University of North Dakota (Approval Number: 2406-0029). A xenograft tumor model of BxPC-3 cancer was established in BALB/c mice (8-10 weeks old, ~20 g, Jackson Laboratories). In brief, BxPC-3 cells suspended in DMEM culture medium (5,000,000 cells/100 μL) were subcutaneously injected into mice. The tumor volume was measured by a digital caliper and calculated using the following formula: Tumor volume = 0.5 × (tumor length) × (tumor width) × (tumor width). Tumor growth was measured every other day. The animals were observed for any signs of pain and changes in body weight every other day throughout the course of the experiments.

In vivo biodistribution

To study the biodistribution of BDP-PTX-NPD and BDP-SS-PTX-NPD in the body, BxPC-3 tumor-bearing mice were allocated randomly into 3 groups, including a control group, 1 group treated with BDP-PTX-NPD, and 1 group treated with BDP-SS-PTX-NPD. Mice were intravenously administered with BDP-PTX-NPD and BDP-SS-PTX-NPD, contained in PBS buffer (pH 7.4) at a dose of 1 mg/kg in the experimental groups and PBS buffer alone in the control group. The mice were euthanized at 24 h post-injection, and their tumors and major organs (heart, liver, spleen, lungs, and kidneys) were excised for ex vivo fluorescence imaging. The fluorescence emission of collected major organs and tumors was measured by a non-invasive Lago X whole-body imaging system (Spectral Instruments Imaging Inc.).

In vivo antitumor efficacy

To study the in vivo antitumor therapeutic efficacy of BDP-PTX-NPD and BDP-SS-PTX-NPD, BxPC-3 tumor-bearing mice were assigned randomly into 6 groups (n = 5 for each group), including sterile PBS + light irradiation (control group), free PTX drug in sterile PBS, BDP-PTX-NPD in sterile PBS, BDP-SS-PTX-NPD in sterile PBS, BDP-PTX-NPD + light irradiation, and BDP-SS-PTX-NPD + light irradiation, with mice individually marked and weighed prior to administration. Mice were intravenously administered the following treatments: sterile PBS buffer (the control group), free PTX at a dose of 15 mg/kg, BDP-PTX-NPD (equivalent to 15 mg/kg of PTX), and BDP-SS-PTX-NPD (equivalent to 15 mg/kg of PTX). The treatments were given to mice every other day. For the groups administered with photodynamic treatment, the tumor sites in mice were irradiated by light at 561 nm with a power of 0.5 W cm^{-2} for 5 min. The volume of tumors in mice was measured every other day. At the end of the experiment, all mice were

euthanized, and the tumors and major organs (heart, liver, spleen, lungs, and kidneys) were excised for further analysis.

Histological analysis

For histological examination, the collected main organs and tumor tissues were fixed in a paraformaldehyde solution (4% in PBS) and then embedded in optimal cutting temperature (OCT) gel. The OCT gels were frozen and sectioned to a thickness of 5 µm, followed by staining with hematoxylin and eosin (H&E) and imaging under a light microscope. The histological analysis was carried out in a blinded manner by professional personnel at the University of North Dakota.

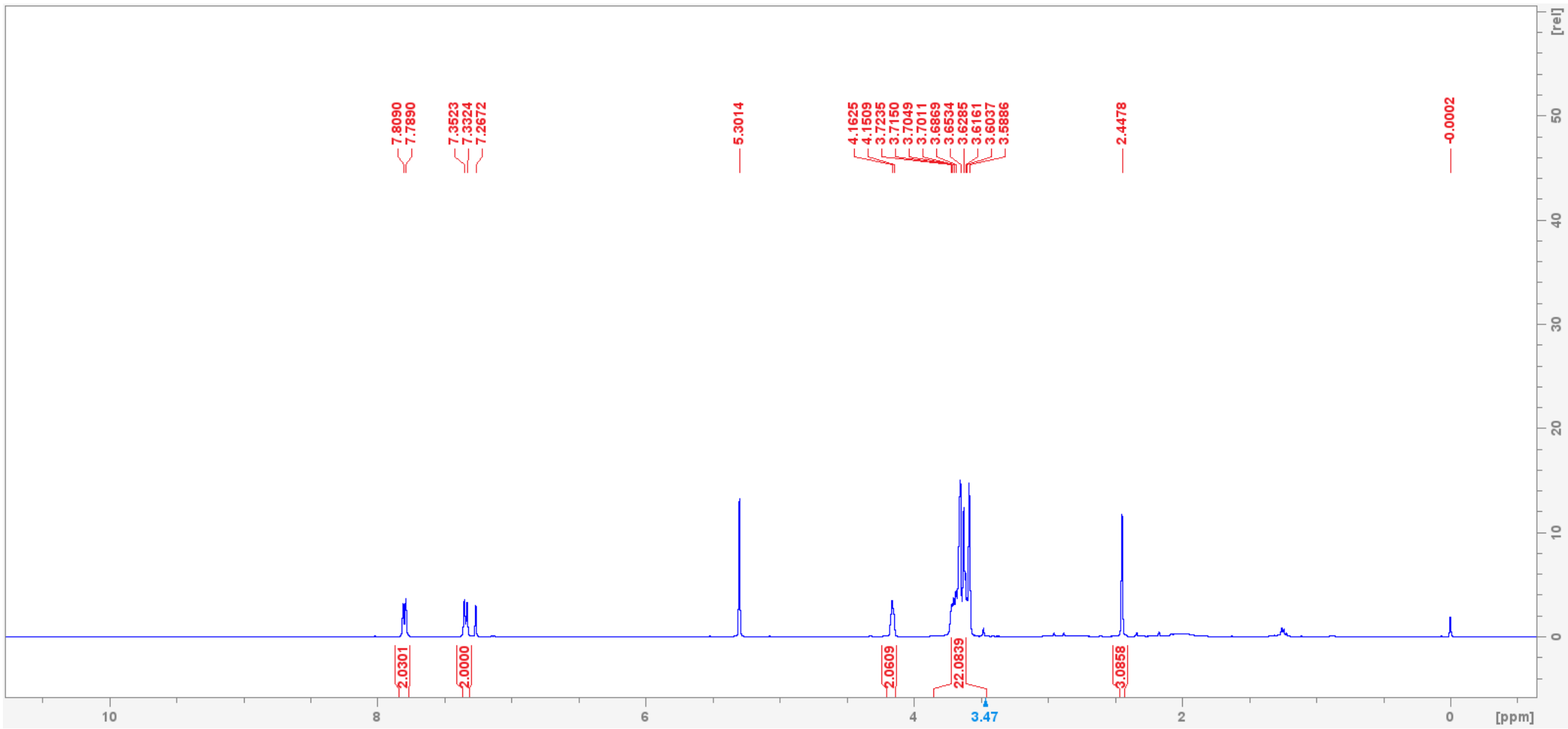

Figure S1. ^{1}H NMR spectrum of compound **1**.

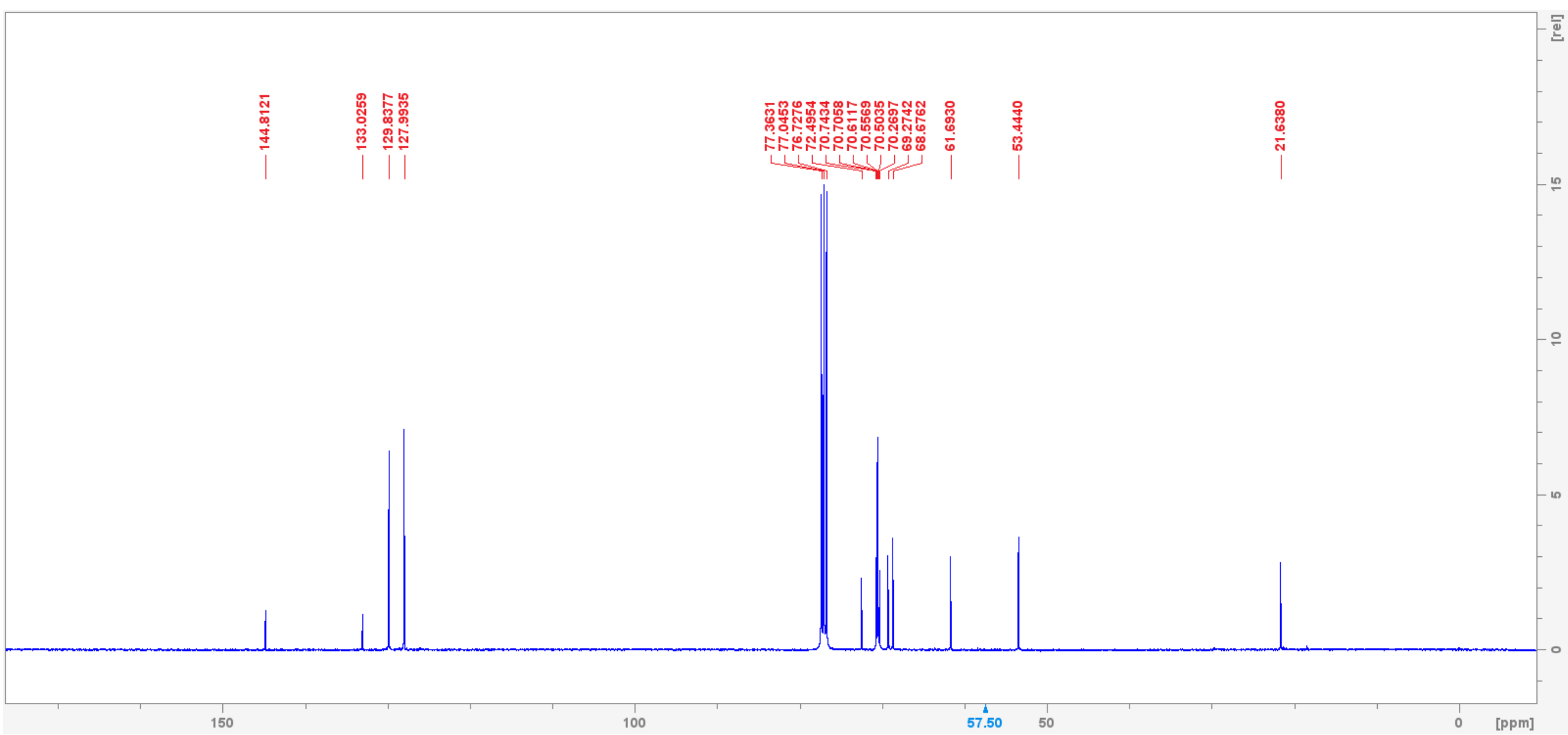

Figure S2. ^{13}C NMR spectrum of compound **1**.

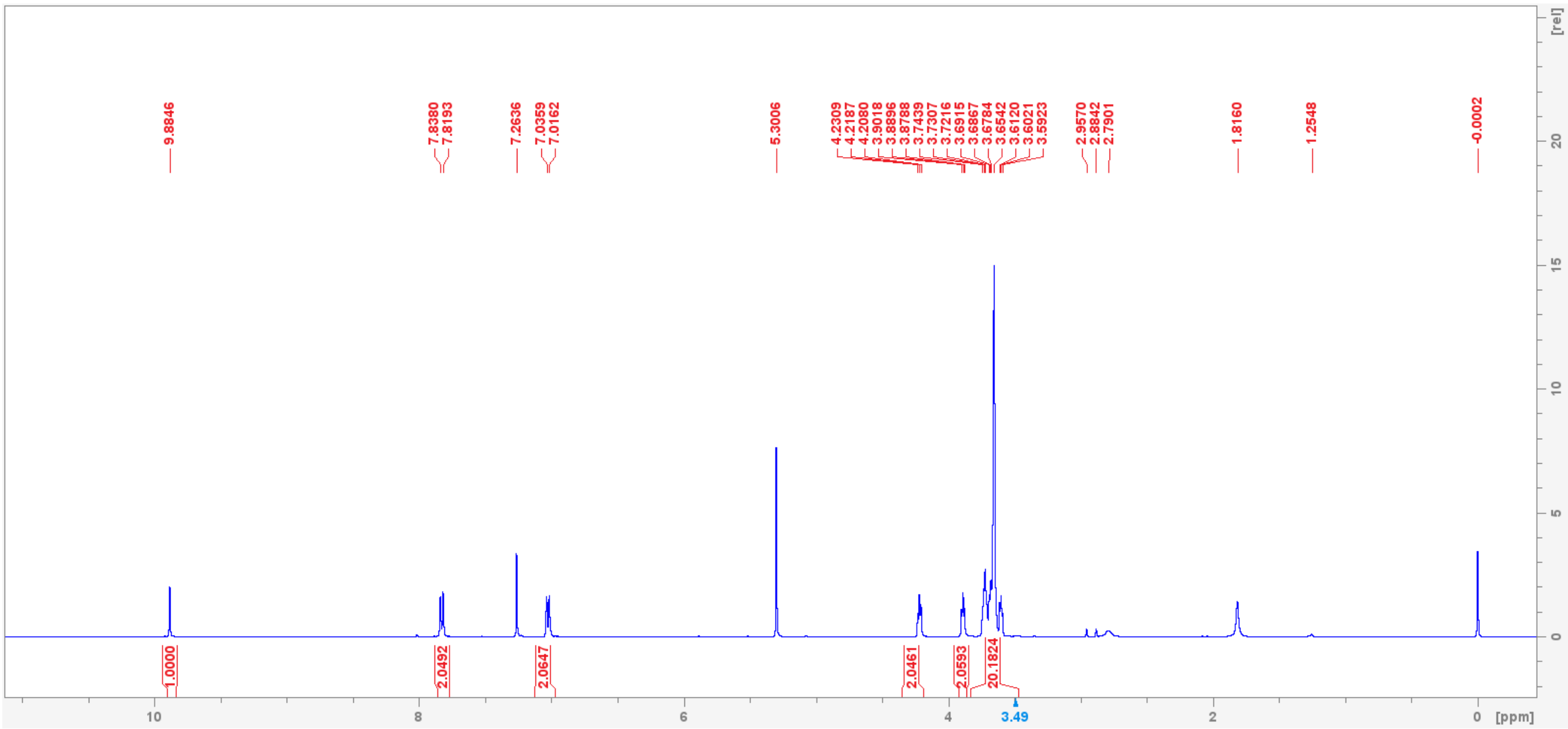

Figure S3. ^{1}H NMR spectrum of compound **2**.

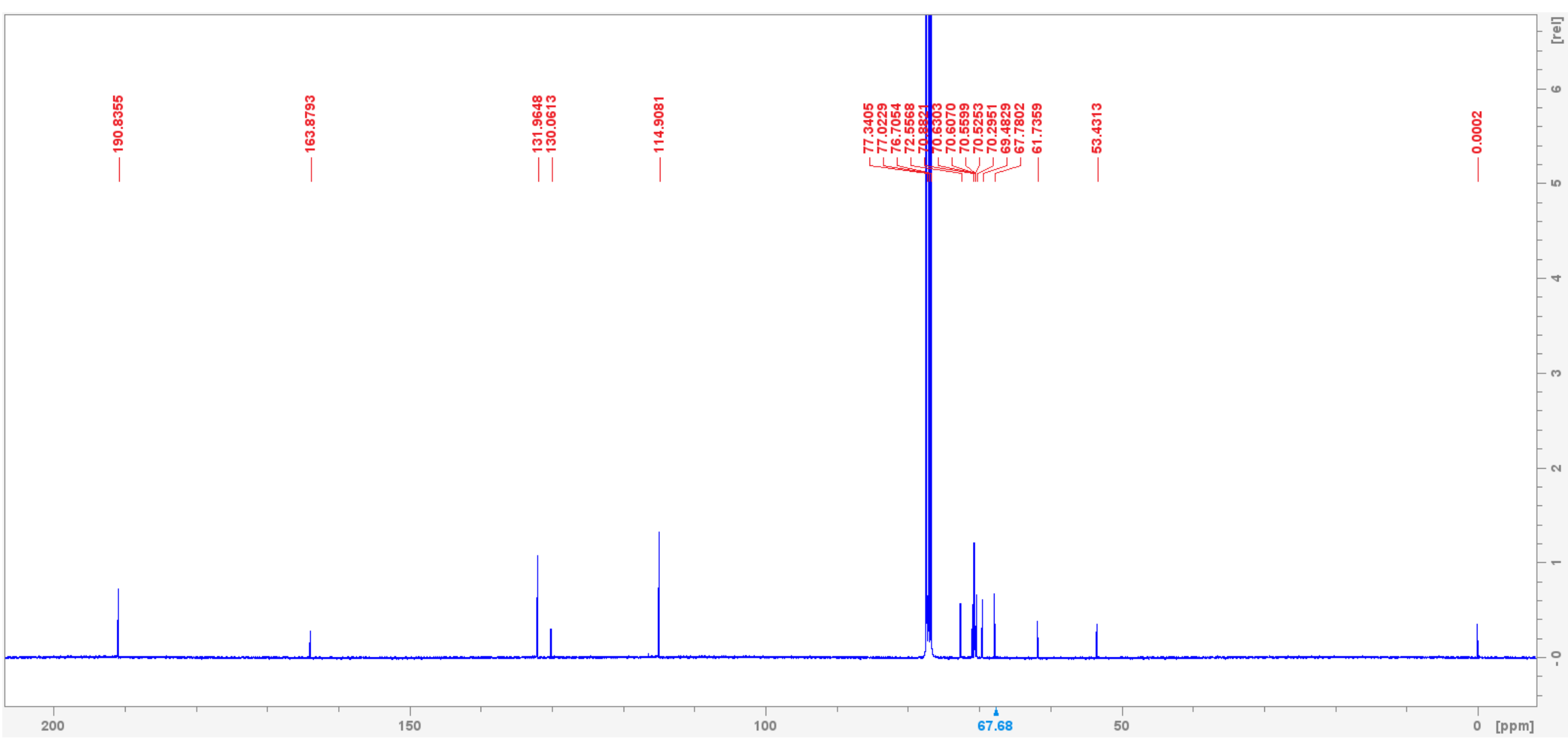

Figure S4. ^{13}C NMR spectrum of compound **2**.

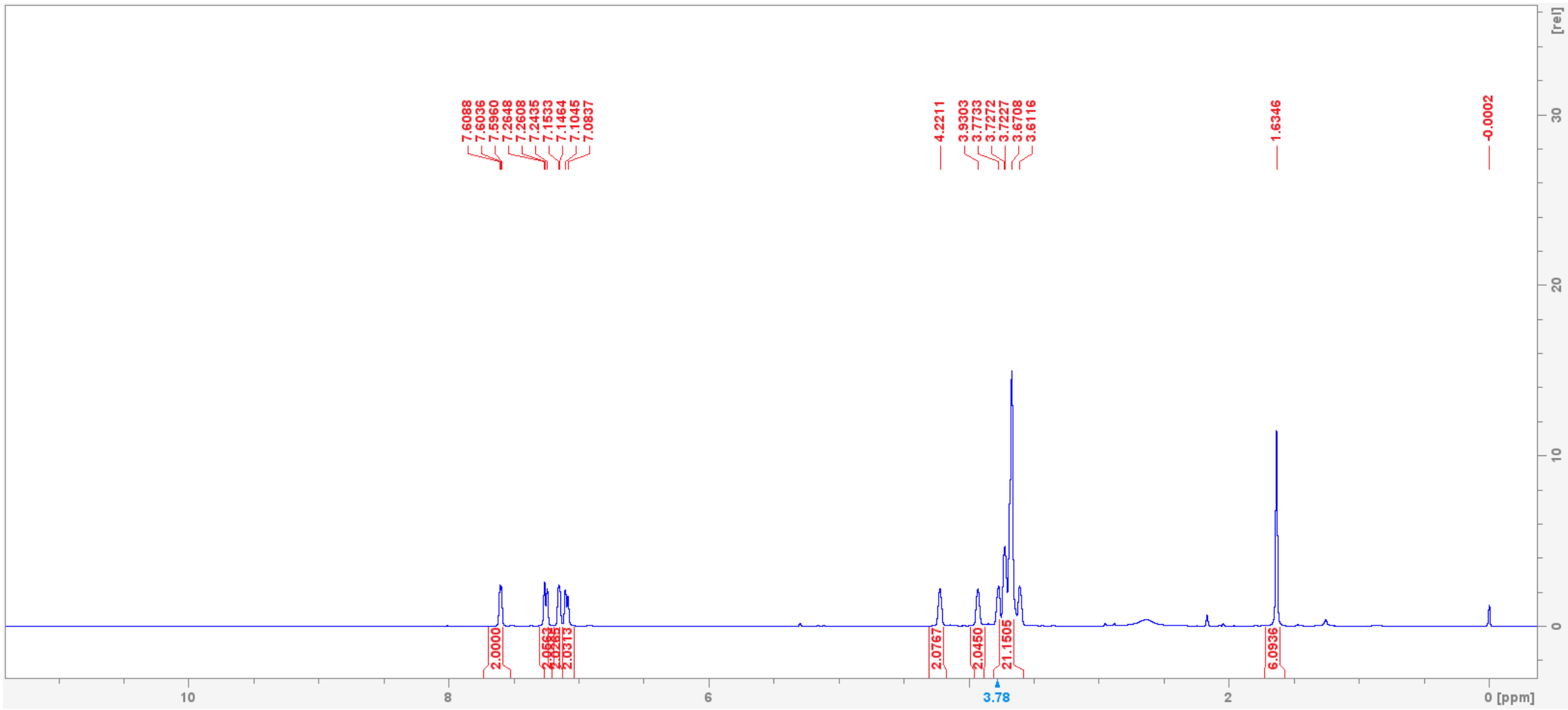

Figure S5. ^{1}H NMR spectrum of BDP-OEG.

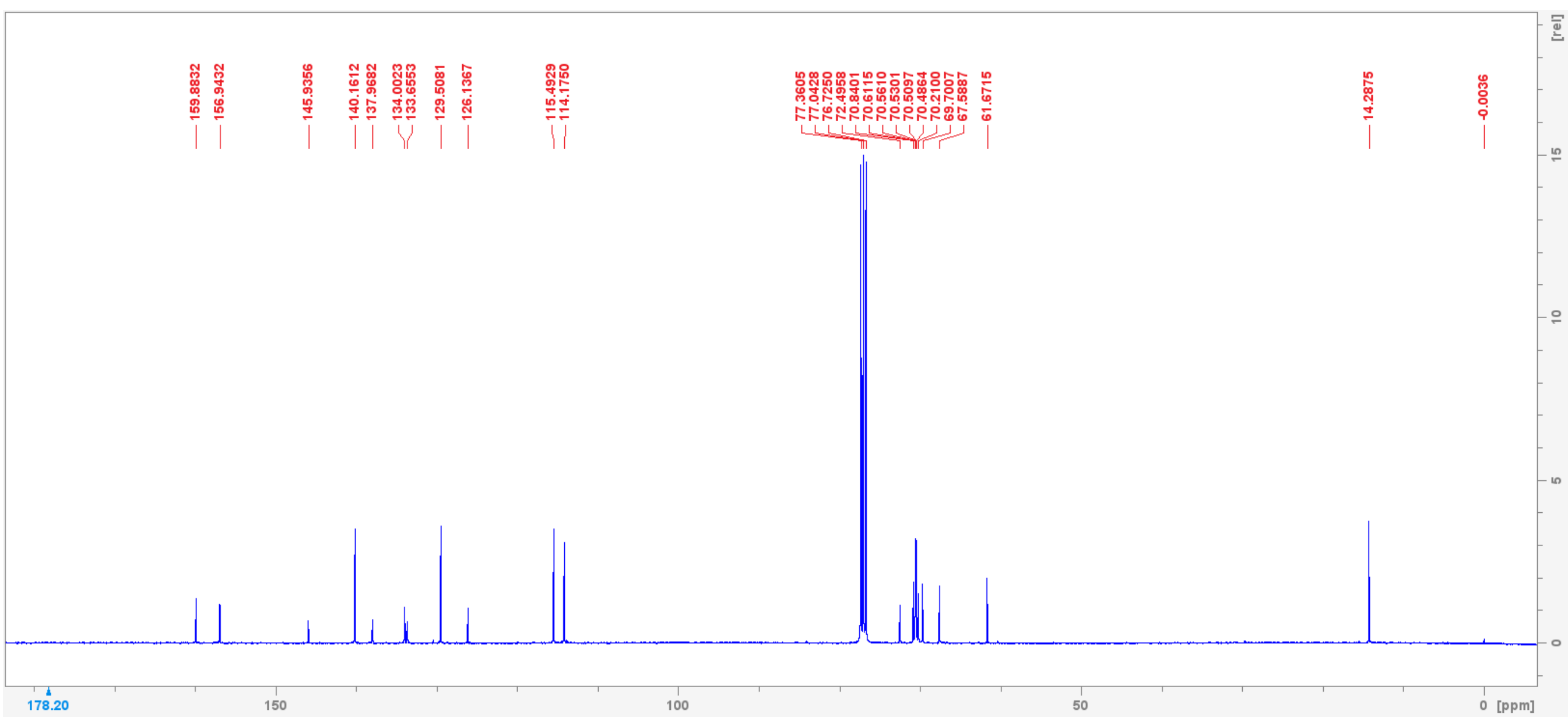

Figure S6. ^{13}C NMR spectrum of BDP-OEG.

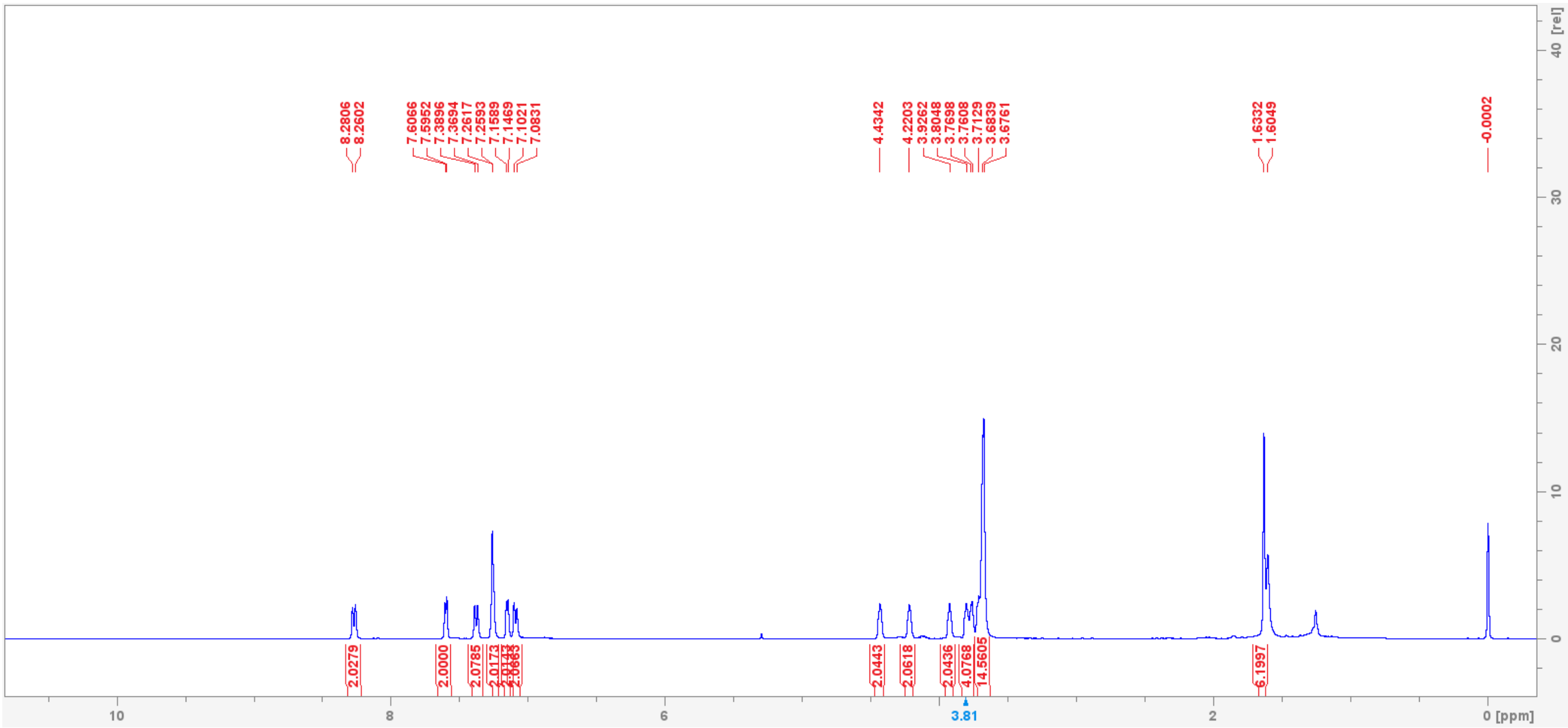

Figure S7. ^{1}H NMR spectrum of BDP-NPC.

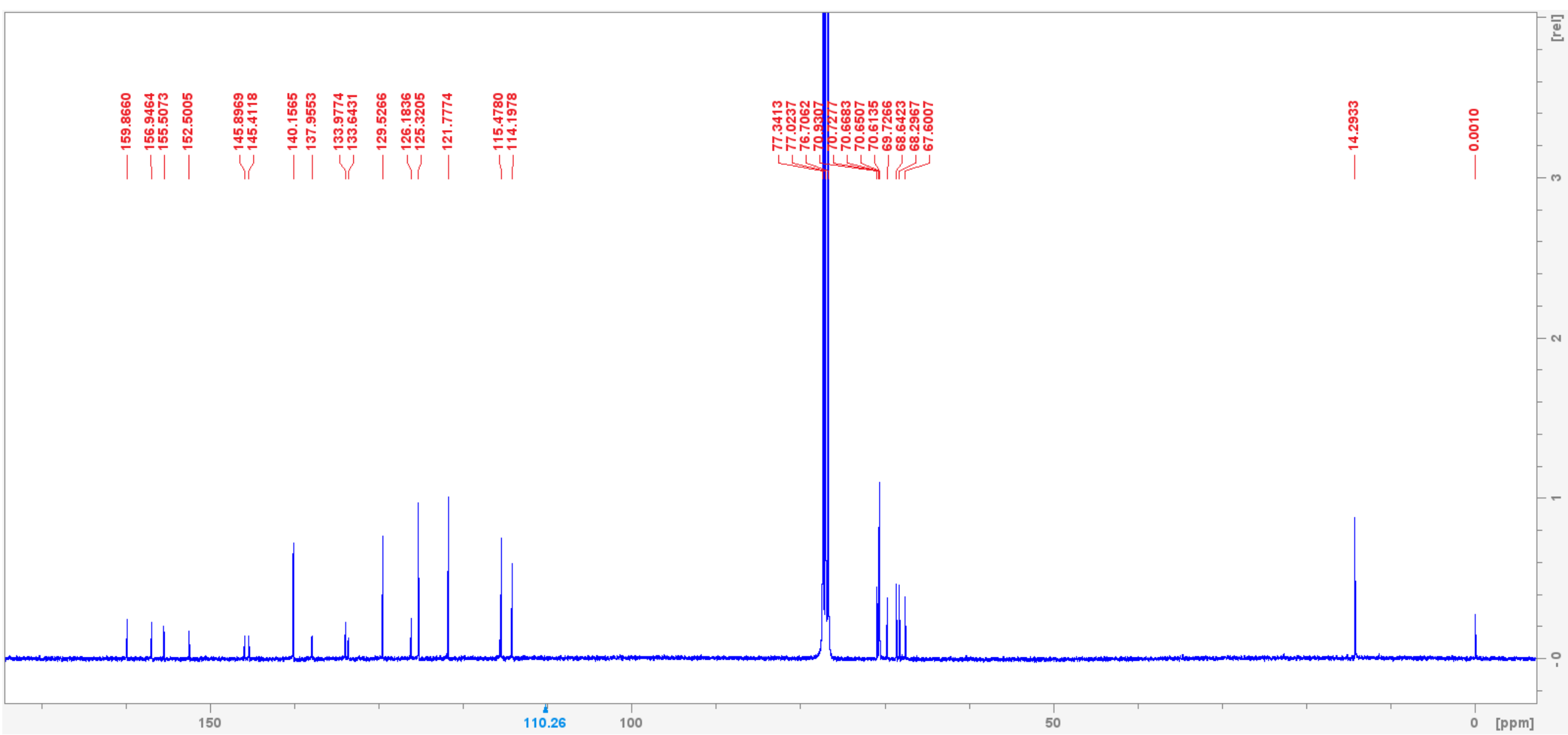

Figure S8. ^{13}C NMR spectrum of BDP-NPC.

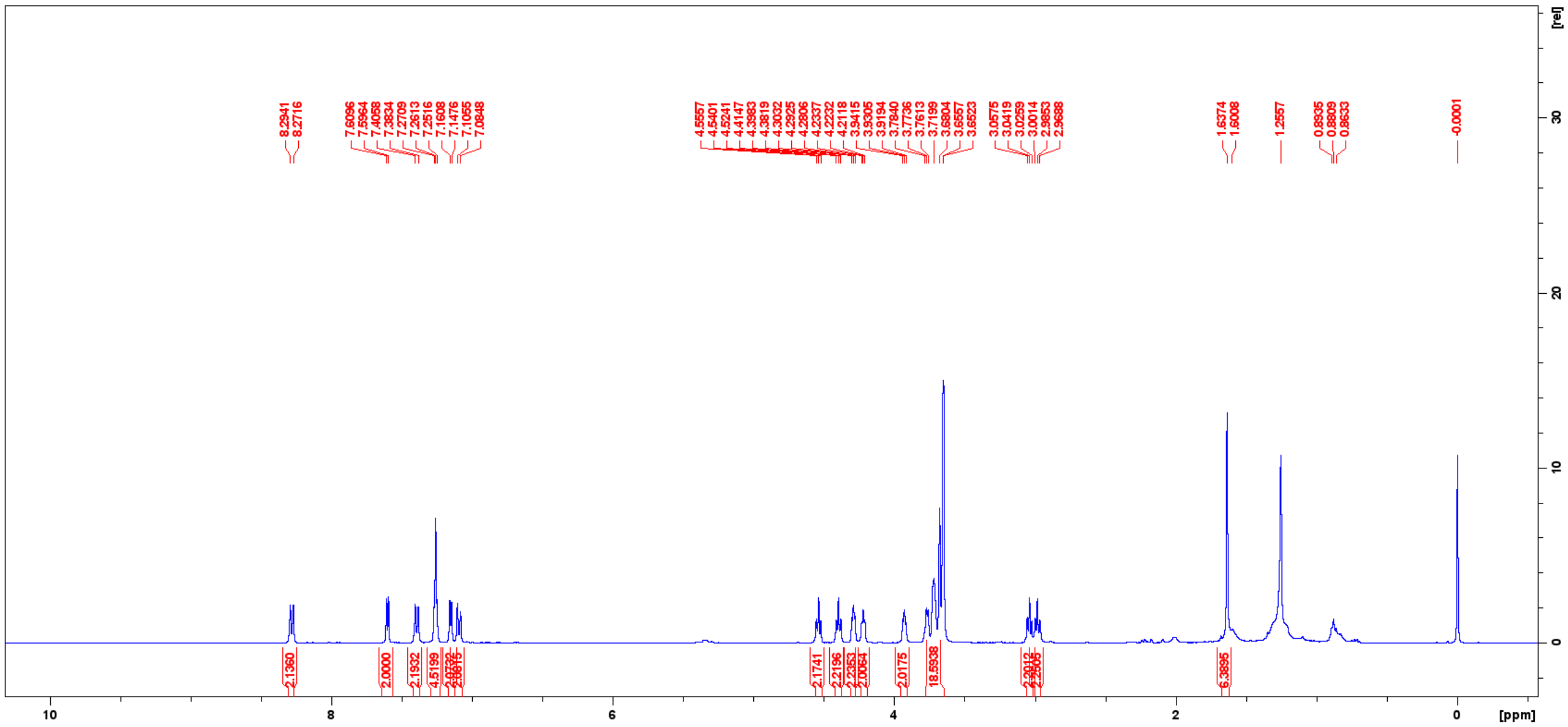

Figure S9. ^{1}H NMR spectrum of BDP-SS-NPC.

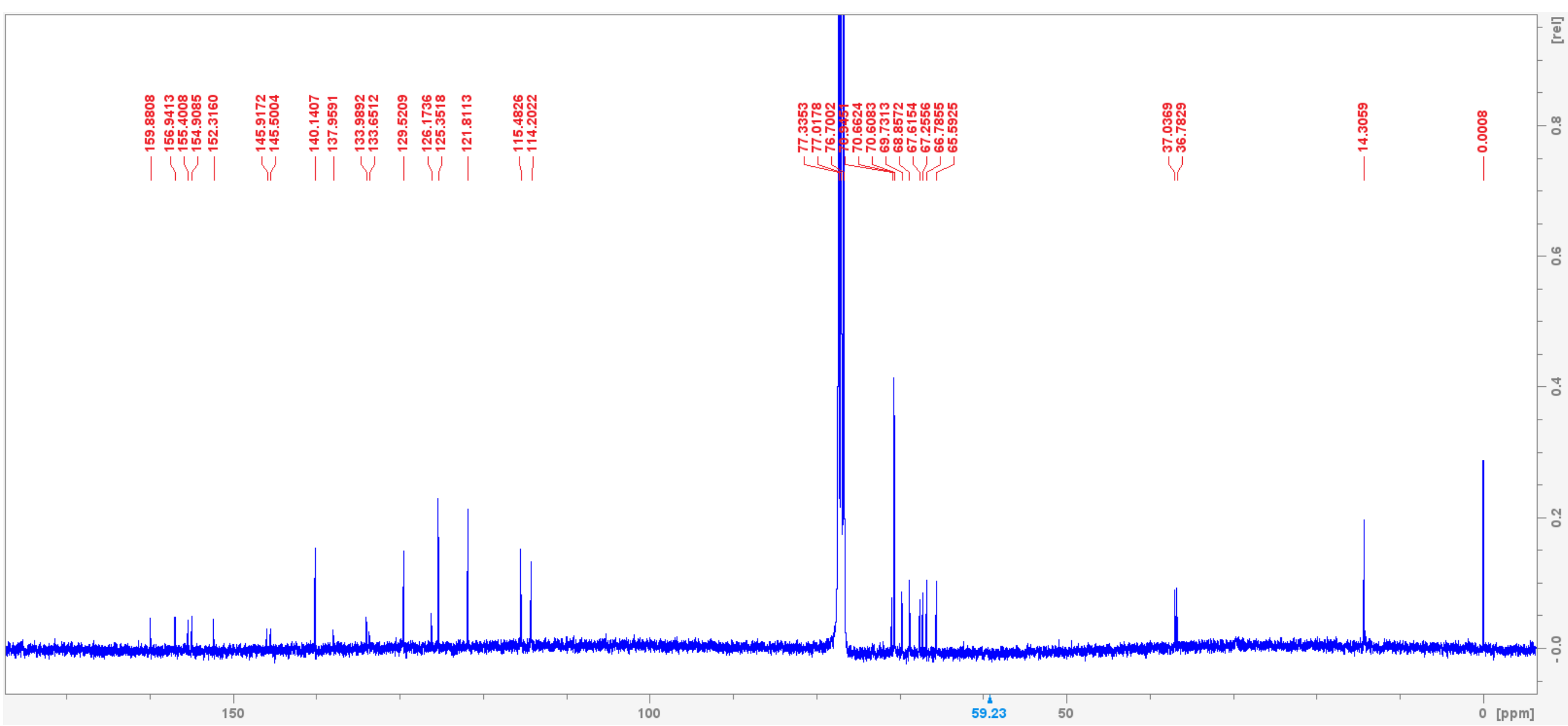

Figure S10. ^{13}C NMR spectrum of BDP-SS-NPC.

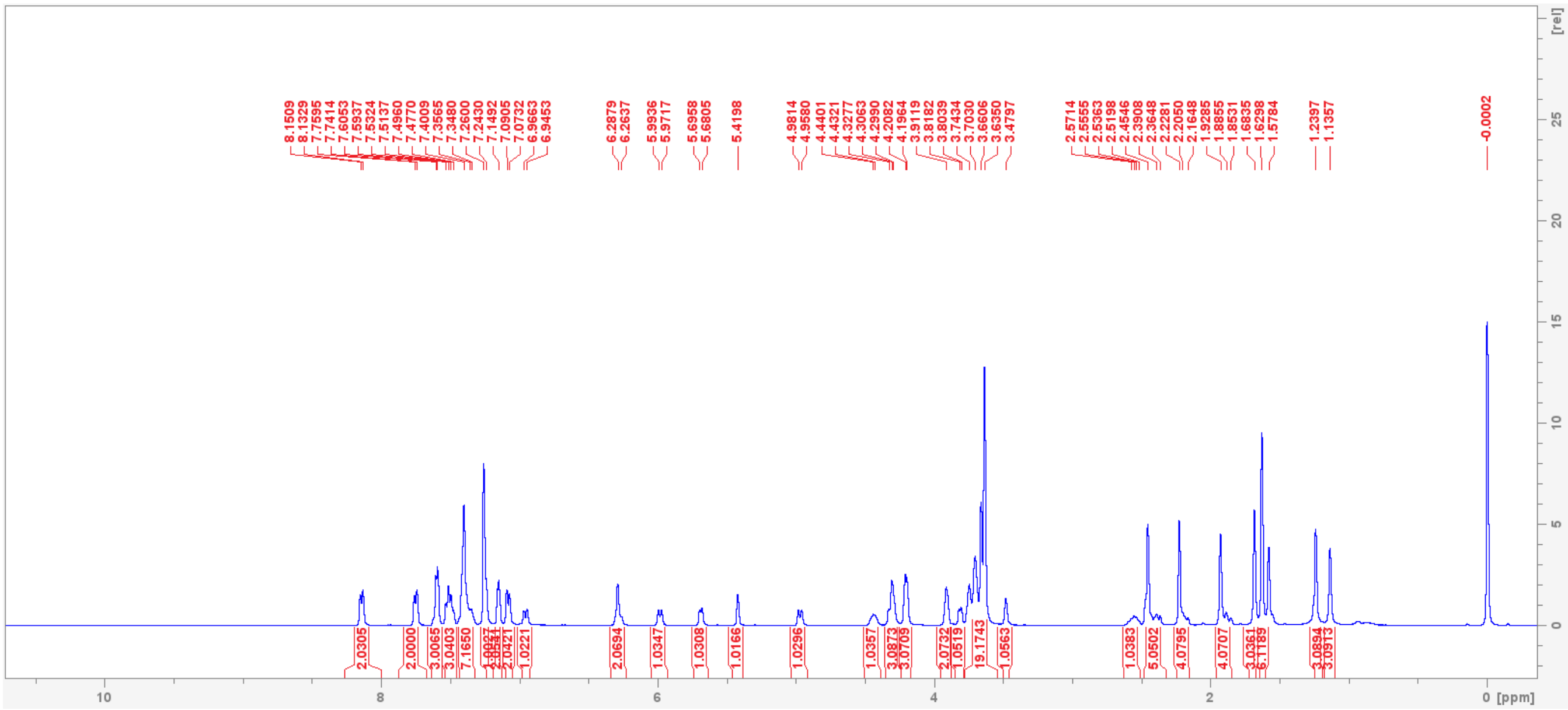

Figure S11. ^{1}H NMR spectrum of BDP-PTX.

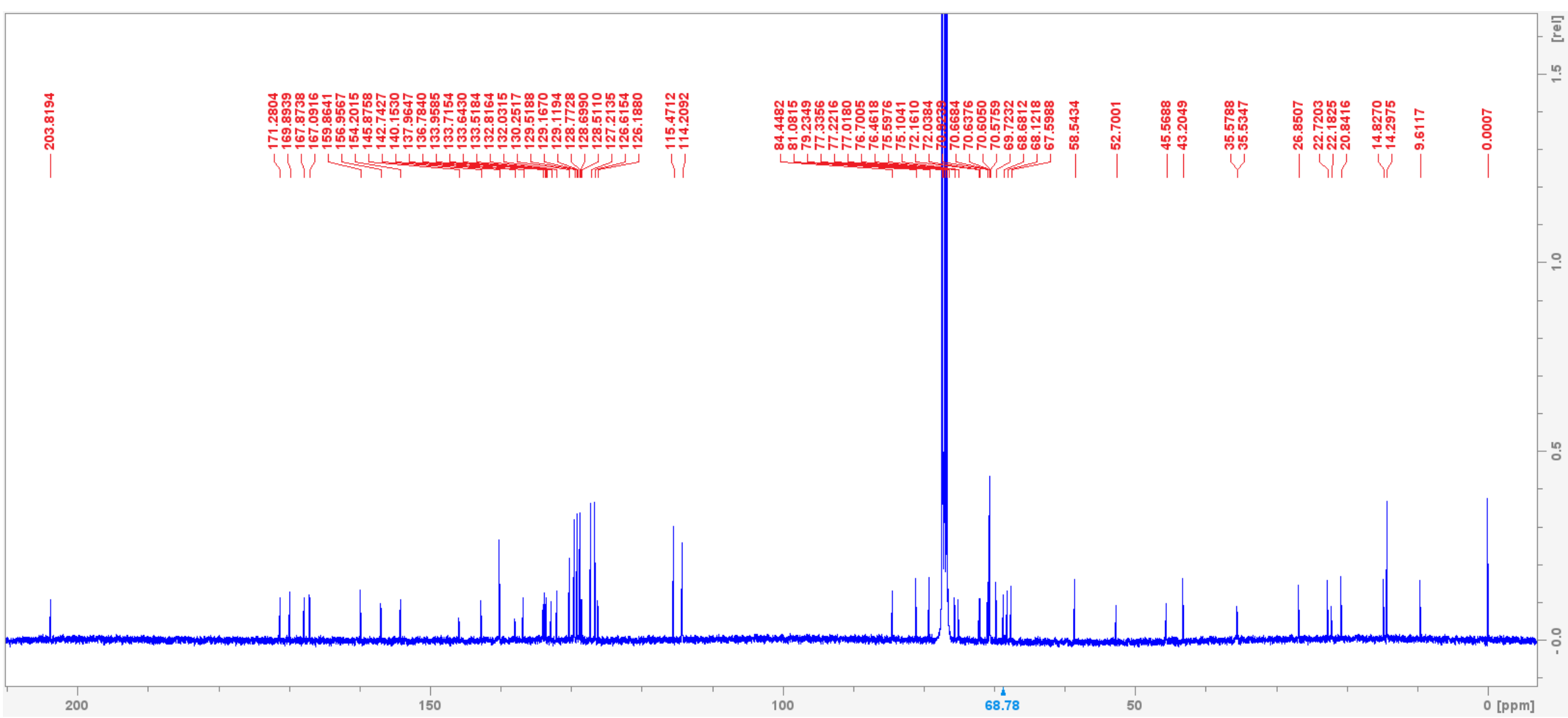

Figure S12. ^{13}C NMR spectrum of BDP-PTX.

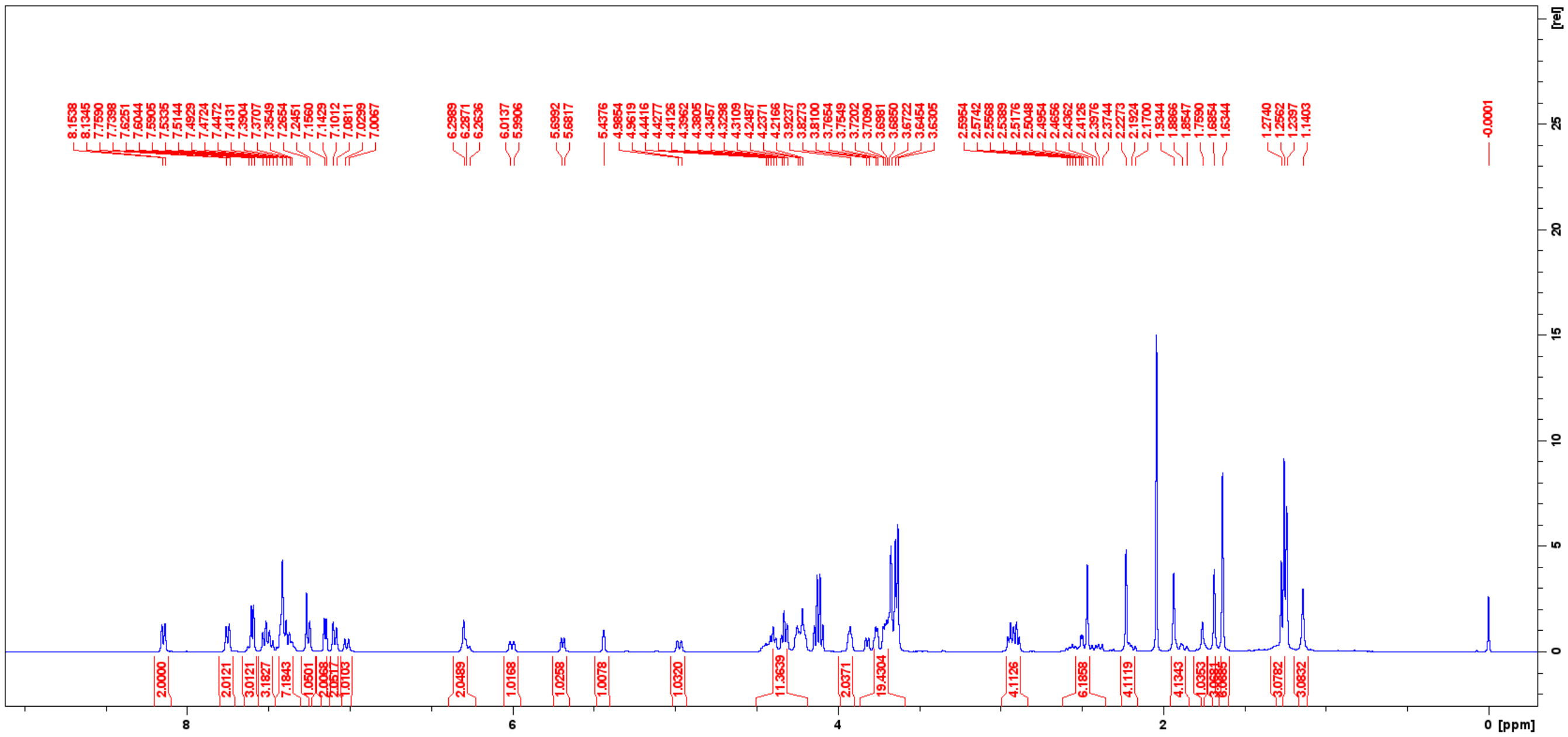

Figure S13. ^{1}H NMR spectrum of BDP-SS-PTX.

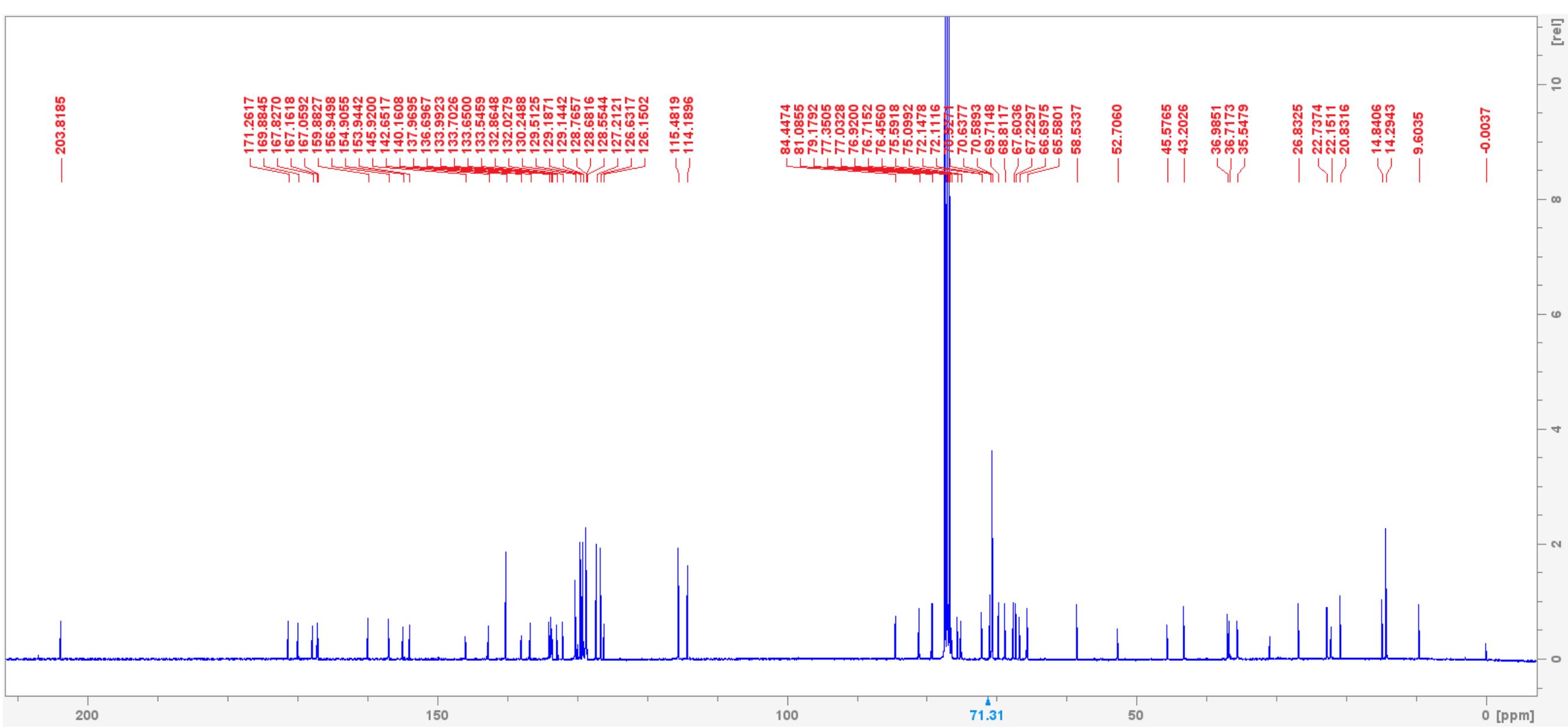

Figure S14. ^{13}C NMR spectrum of BDP-SS-PTX.

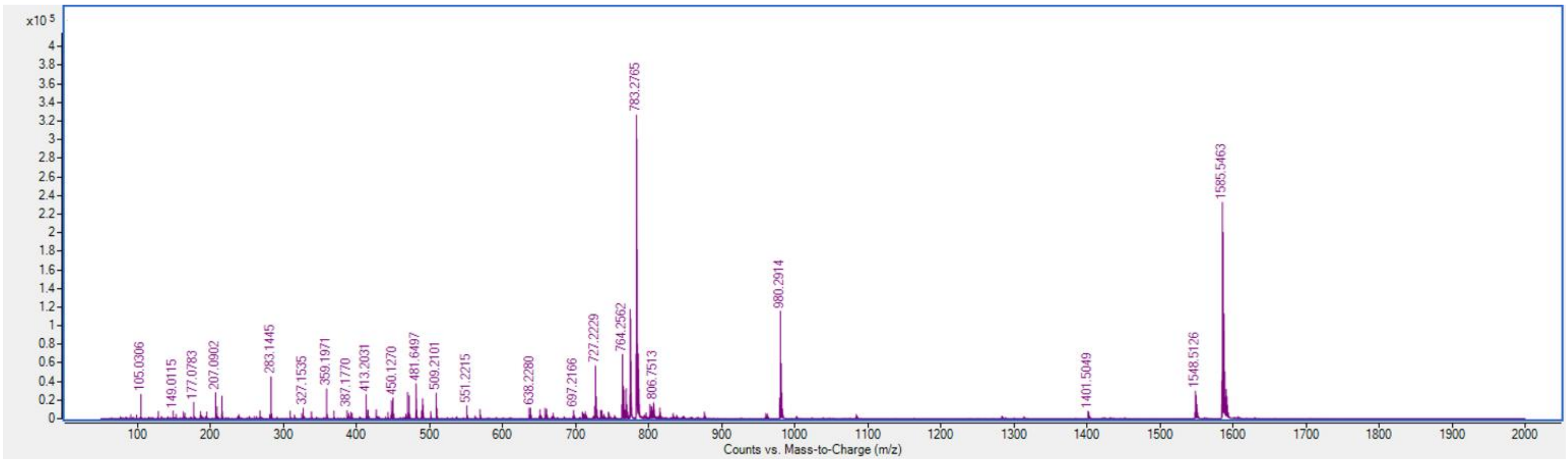

Figure S15. HRMS (ESI, m/z) spectrum of BDP-PTX. $C_{81}H_{88}BF_2N_3O_{22}S_2$ ($[M+NH_4]^+$): calcd 1585.5706; found 1585.5463.

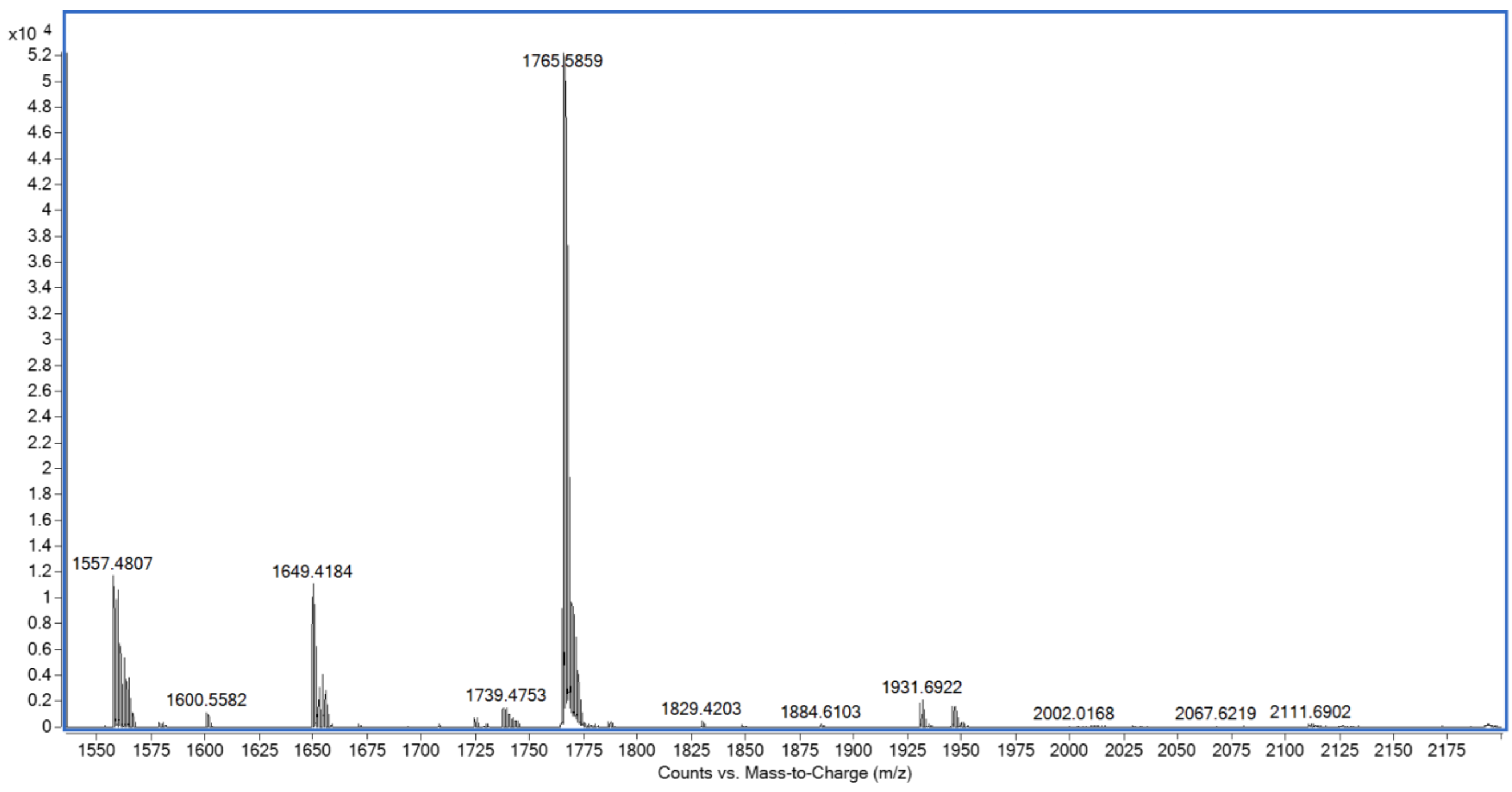

Figure S16. HRMS (ESI, m/z) spectrum of BDP-SS-PTX. $C_{86}H_{96}BF_2N_3O_{25}S_4$ ($[M+NH_4]^+$): calcd 1765.5621; found 1765.5859.

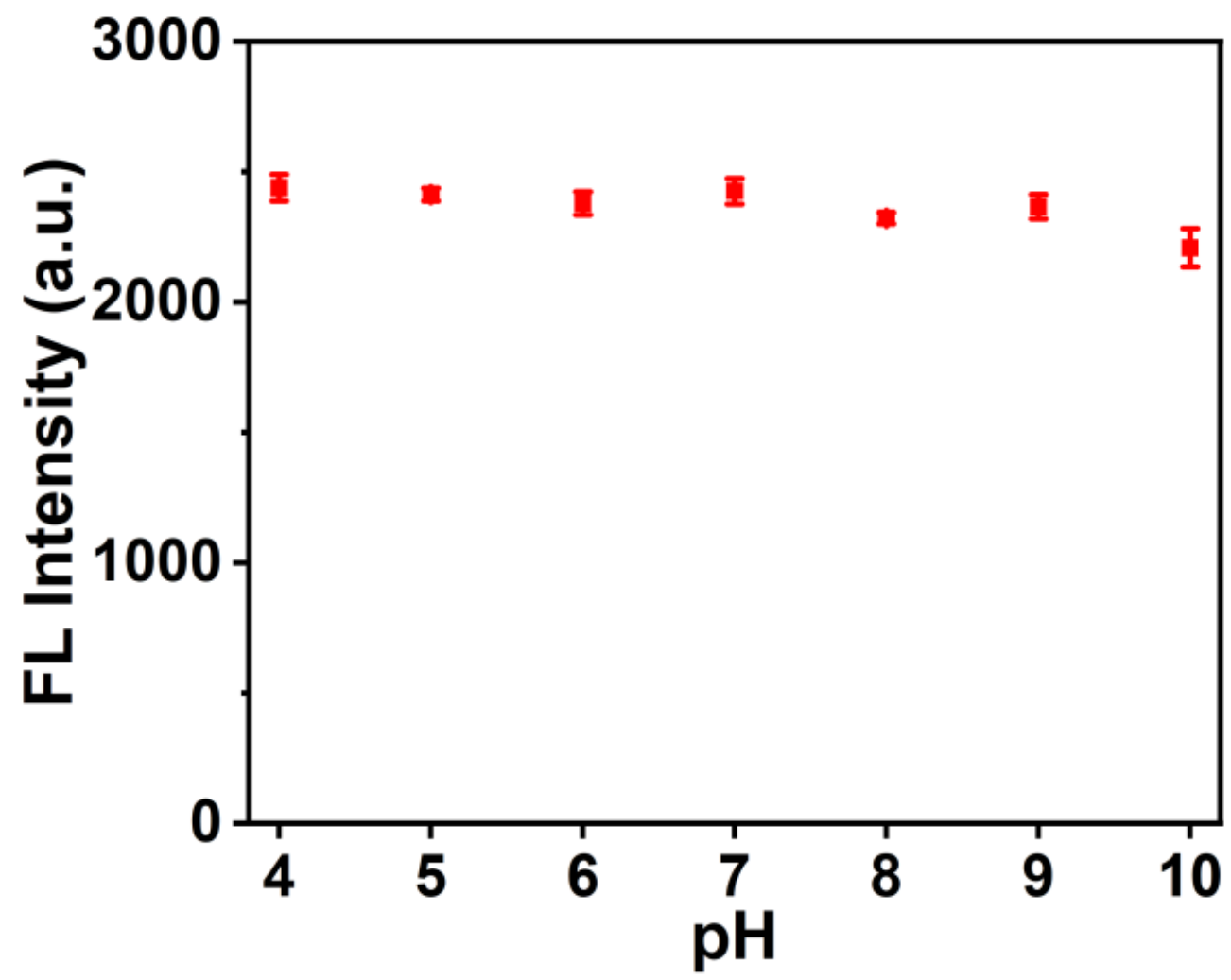

Figure S17. Fluorescence emission intensity of BDP-SS-PTX (5.0 μM) at varied pH.

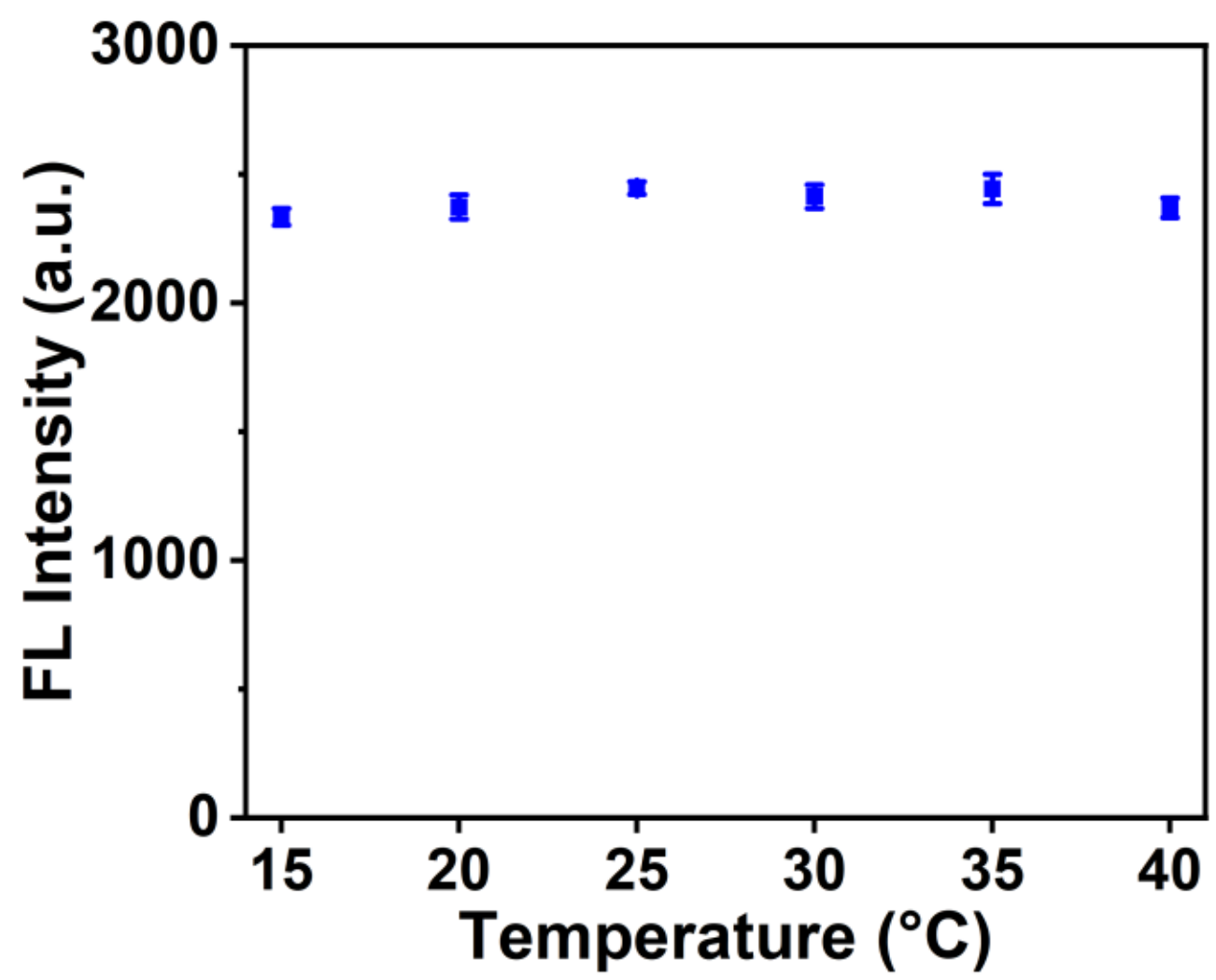

Figure S18. Fluorescence emission intensity of BDP-SS-PTX (5.0 μM) at varied temperatures.

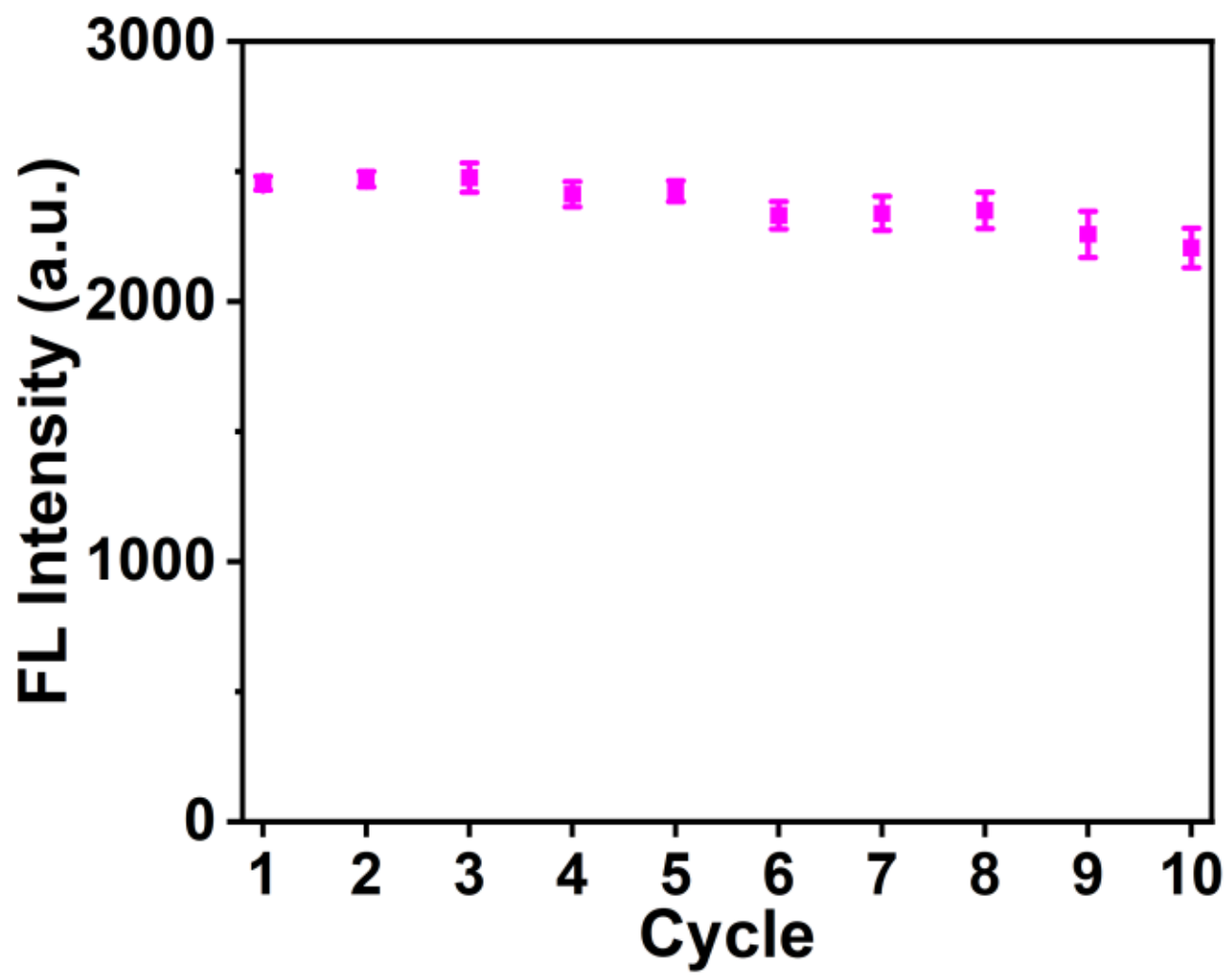

Figure S19. Fluorescence emission intensity of BDP-SS-PTX (5.0 μM) after 10 repeated excitation cycles.

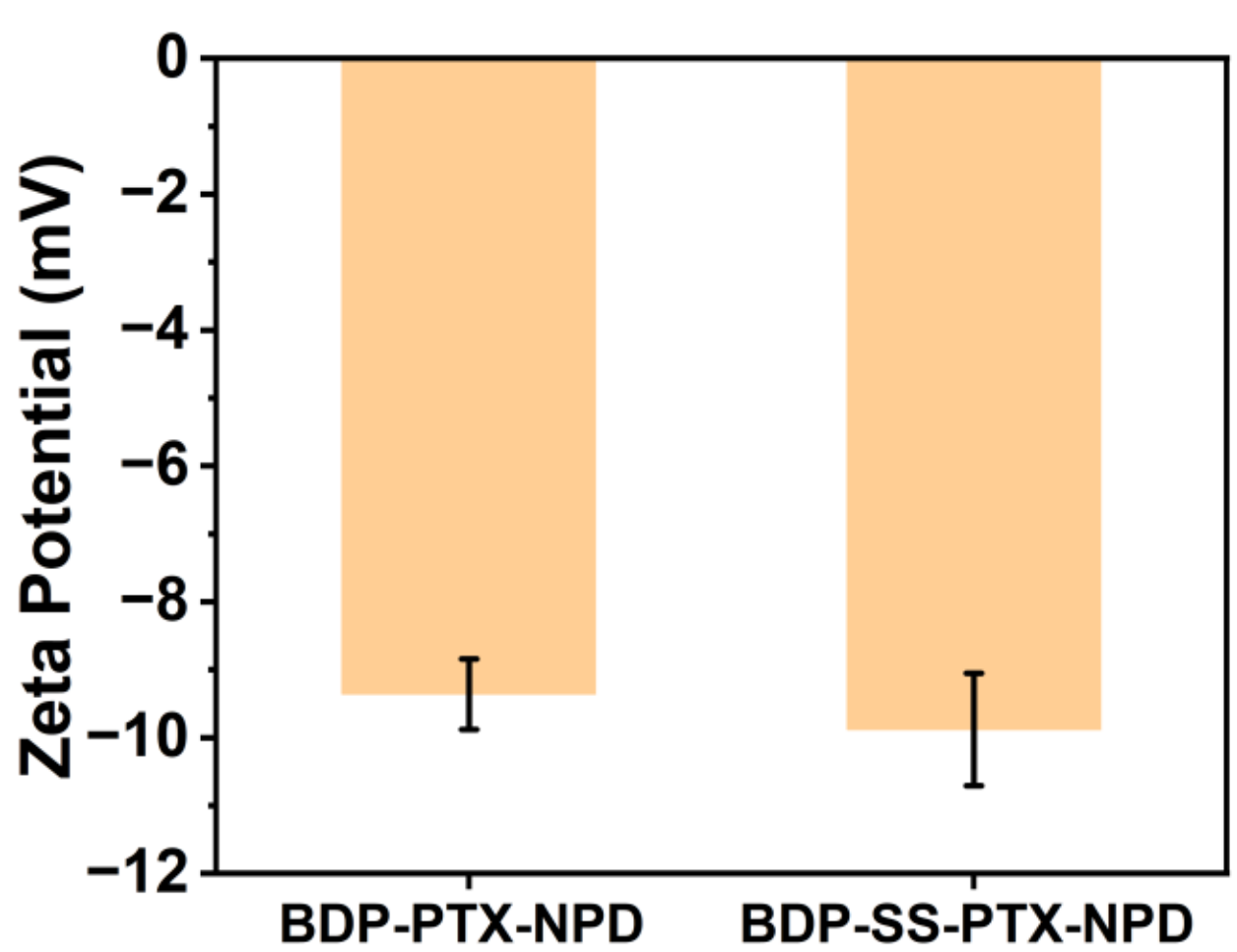

Figure S20. The zeta potential of BDP-PTX-NPD and BDP-SS-PTX-NPD.

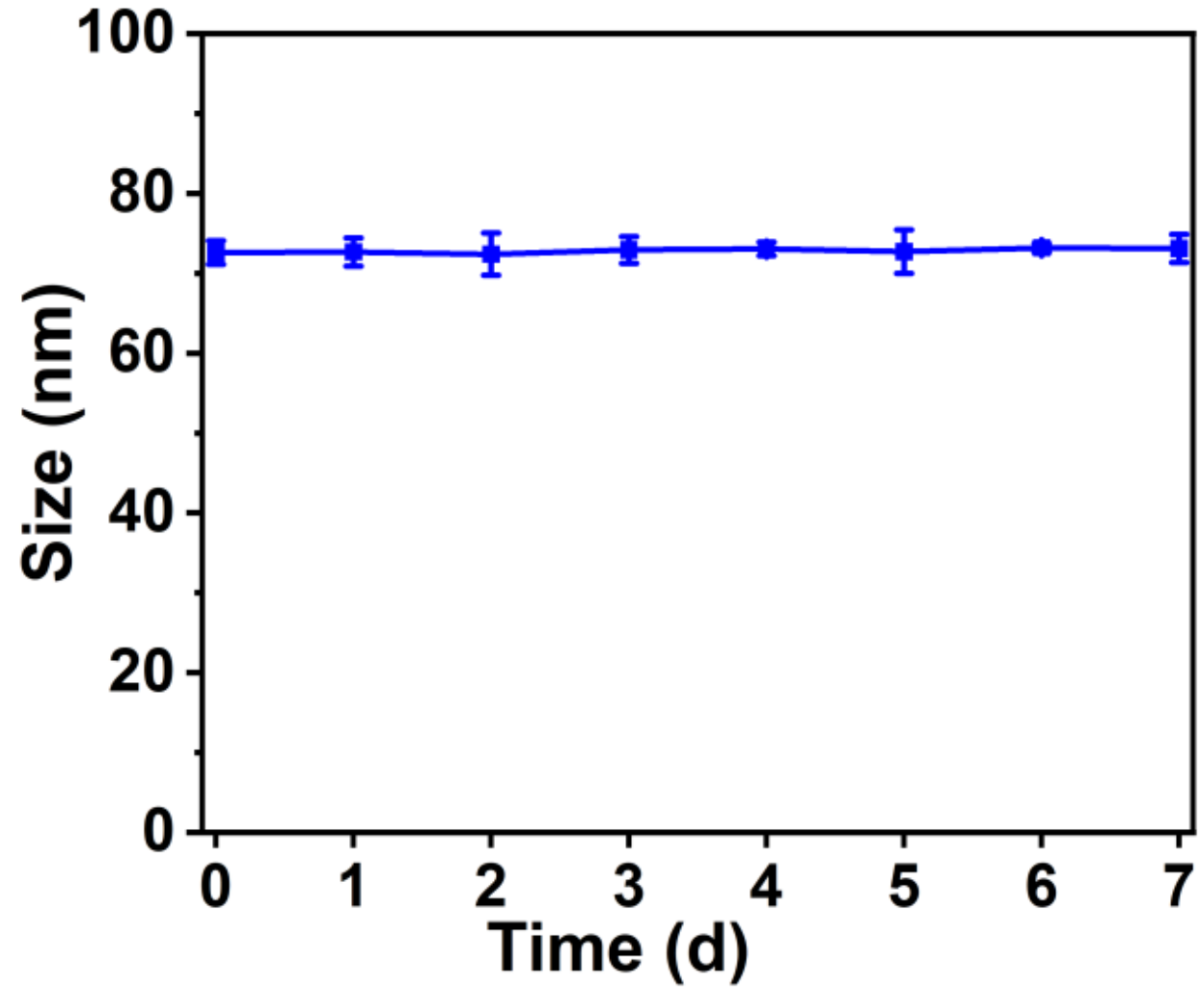

Figure S21. The hydrodynamic size of BDP-SS-PTX-NPD in DI water over a week.

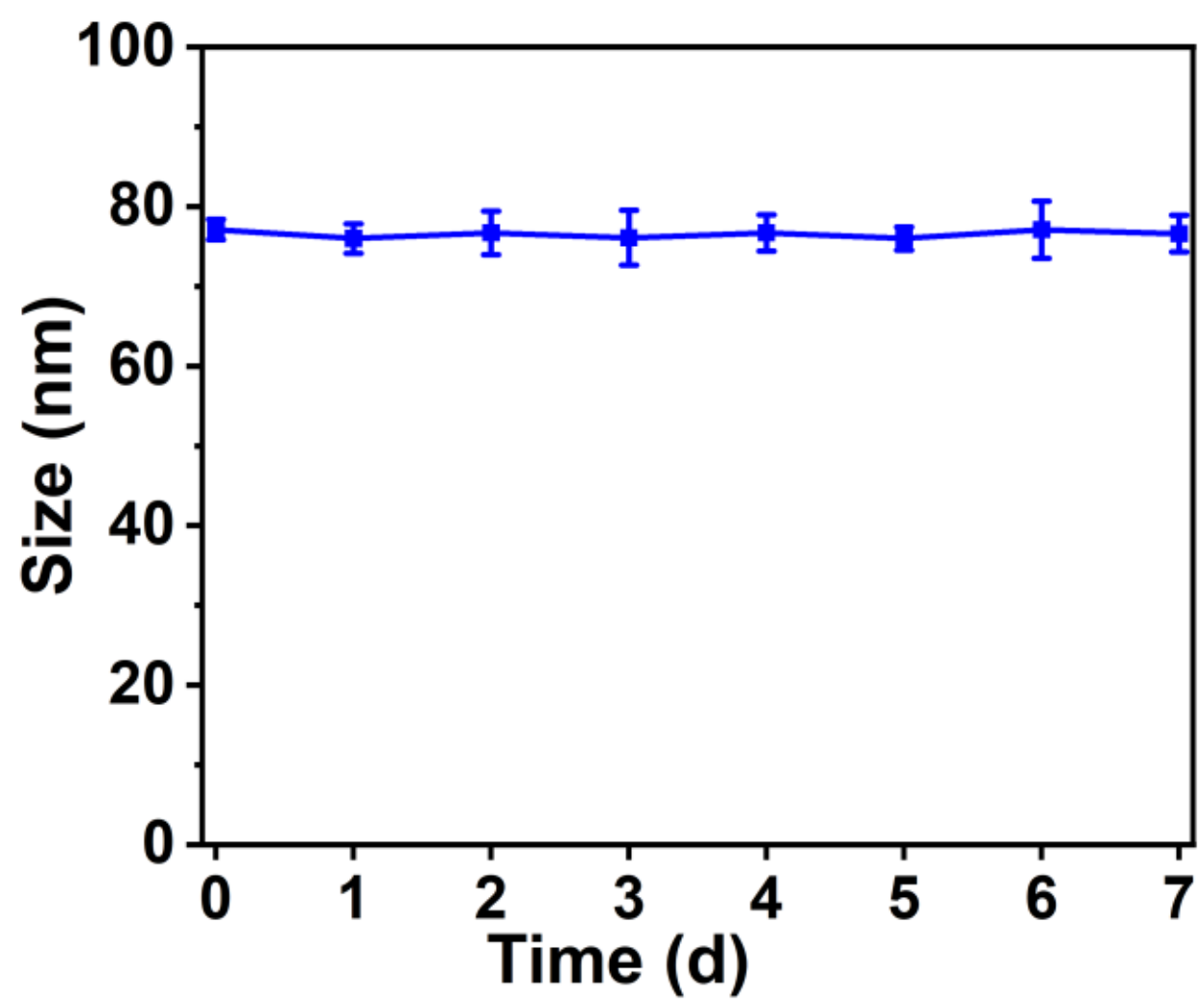

Figure S22. The hydrodynamic size of BDP-PTX-NPD in DI water over a week.

References

1. Sujie Qi, Nahyun Kwon, Yubin Yim, Van-Nghia Nguyen, and Juyoung Yoon. Fine-tuning the electronic structure of heavy-atom-free BODIPY photosensitizers for fluorescence imaging and mitochondria-targeted photodynamic therapy. *Chem. Sci.* **2020**, *11*, 6479-6484.

2. Safiya Nisar, Elisabeth Starosta, Mouhmad Elayyan, Amrit Regmi, and Binglin Sui. Photoinduced Electron Transfer-Based Glutathione-Sensing Theranostic Nanoprodrug with Self-Tracking and Real-Time Drug Release Monitoring for Cancer Treatment. *ACS Appl. Mater. Interfaces.* **2024**, *16*, 6859-6867.

3. Lakowicz, J. R., *Principles of fluorescence spectroscopy*. 3rd ed.; Springer: New York, 2006; p xxvi, 954 p.

4. Ting-Chao Chou. Theoretical basis, experimental design, and computerized simulation of synergism and antagonism in drug combination studies. *Pharmacol. Rev.* **2006**, *58*, 621-681.
